# Developing *SCL2205*: a protein sequence-based spatial modelling dataset for the protein language model frontier

**DOI:** 10.1093/bioinformatics/btag666

**Published:** 2026-09-18

**Authors:** Daniel Ouso, Gianluca Pollastri

**Affiliations:** School of Computer Science, University College Dublin - Belfield, Dublin D04 N2E5, Ireland; Centre for Research Training in Genomics Data Science, University of Galway, Galway H91 TK33, Ireland; School of Computer Science, University College Dublin - Belfield, Dublin D04 N2E5, Ireland

## Abstract

**Motivation:**

Deep learning (DL) has substantially advanced protein subcellular localization (SCL) prediction, yet its potential remains constrained by suboptimal input preparation and limited high-quality reference data. Furthermore, existing state-of-the-art (SoTA) predictors suffer from performance metric inflation due to unmitigated training-to-testing data leakage during homology augmentation. We address these challenges by introducing *SCL2205*, a leak-minimized benchmark dataset and pipeline curated specifically to support trustworthy, scalable, and reproducible DL-based SCL modelling.

**Results:**

*SCL2205* was constructed from the universal protein knowledgebase (UniProtKB) using rigorous preprocessing, manual label mapping, and stringent partitioning. When evaluated on independent test sets, *SCL2205* yielded up to a 10.8 percentage point improvement in macro area under the precision–recall curve (PR-AUC) over SoTA baselines (*mean* Δ 95% CI = 0.07–0.12), with maximum benefits observed when paired with modern protein language models (PLMs). Crucially, we quantify for the first time a systemic 5.2%±0.32 data leakage rate in conventional homology augmentation workflows—even when restricting sequence similarity searches to just 10% of the training set.

**Availability and implementation:**

The dataset is openly available on Dryad under a CC0 1.0 Universal licence (https://doi.org/10.5061/dryad.2ngf1vj1t). The dataset interface is available as an installable Python package, p-scldata (v2026.2.0), under the MIT licence on the Python Package Index (PyPI). Full code and data repositories are hosted on GitHub (https://github.com/ousodaniel/scldata) and archived on Zenodo (https://doi.org/10.5281/zenodo.21796423).

## 1 Introduction

Automated sequence annotation is a critical component of functional genomics. It is mainly driven by the widespread penetration of next-generation sequencing. The adoption of artificial intelligence (AI) for protein subcellular location annotation remains an active research area currently dominated by sequence-based deep learning (DL) predictors. Owing to its ability to model complex phenomena and directly exploit sequence information, DL has become more popular than classical machine learning (ML). The latter approach relies on pre-determined sequence characteristics as modelling features. However, sufficient quality training data are the main bottleneck in maximizing supervised DL predictors, which are very data-intensive. Regardless of the data challenge, it is paramount to provide high-quality training input.

Although universal protein knowledgebase (UniProtKB) is a primary source of sequences for subcellular localization (SCL) DL predictors, researchers often prepare their data differently, introducing avoidable bias. These biases may compromise the fair and accurate evaluation of different predictors. Moreover, suboptimal handling of intrinsic yet problematic data characteristics, such as sequence homology, can lead to costly consequences, such as training-to-testing data overlap. In addition, some predictors continue to rely on outdated database versions, despite significant improvements in data quality and quantity. This is likely associated with the rigorous and time-consuming nature of data development.

Notable advancements in AI have been driven by well-curated datasets across domains, including computer vision (LeCun and Cortes 2010), natural language processing (Maas *et al.* 2011), and protein structure prediction—e.g. the Critical Assessment of Structure Prediction datasets. In the SCL domain, the DeepLoc dataset (Armenteros *et al.* 2017) is well known, alongside others such as MultiLoc (Höglund *et al.* 2006). In DeepLoc, sequences were filtered to include only eukaryotic, non-fragment, nucleus-encoded proteins, with a minimum length of 41 amino acids and confirmed experimental annotations (Armenteros *et al.* 2017, Thumuluri *et al.* 2022). In contrast, MultiLoc (Höglund *et al.* 2006, Blum *et al.* 2009) applies filters based on eukaryotic origin and selected keywords from sequence comments and features. Given the processing disparities, which make performance comparisons problematic, it would be desirable to standardize and consistently curate the input data across all predictors.

As concerns arise about the sustainability and environmental costs of training DL-based AI, the need for high-quality training input is critical to mitigate noisy data, which inflate training costs. Consequently, recent research has demonstrated gains with smaller yet well-curated training datasets in leading domains of AI such as large language models (LLMs) (Marion *et al.* 2023). On the other hand, trustworthiness and the precision of the performance report in AI research are common concerns, especially in critical sectors such as biology and health. Similarly, compliance efforts are proposed for biological AI modelling (Walsh *et al.* 2021). In light of the above, we set out to develop a robust SCL dataset for DL predictor modelling, whilst underscoring current challenges and positioning for emerging frontiers. Beyond common preprocessing practices, such as filtering, we make the following major contributions with our dataset.

- We highlight the limitations of homology augmentation by affirming and, for the first time, quantifying its contribution to data leakage (Whalen *et al*. 2021) using metrics comparable to those used in homology reduction, thereby underscoring trustworthiness in predictors.
- Unlike in other cases, we perform extensive and robust homology reduction by minimizing training-to-validation and training-to-testing across-set overlap to ≤30% homology, while preserving the sequence-length distribution of the original dataset.
- We provide two streams for the dataset—*SCL2205—*for model training: (a) a *training-validation* split and (b) a *5-fold cross-validation* set. In addition, a *held-out* independent testing set is provided for final evaluation.
- We use domain knowledge to manually map labels to spatially higher-level subcellular location (SL) commonly used with SCL predictors.

**Figure 1:**
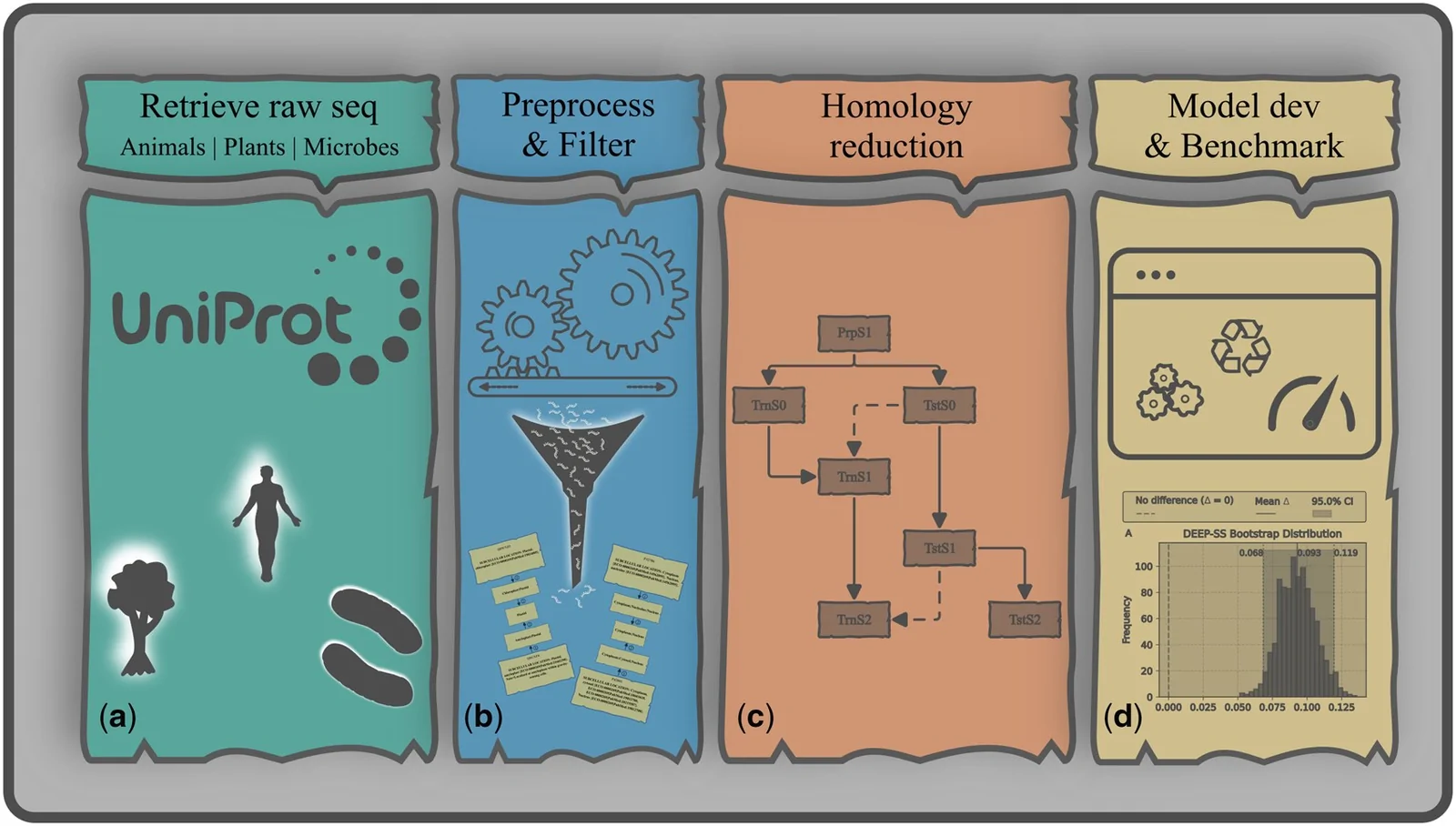
Study graphical summary. **(a)** Source data acquisition, constituting proteins from the major domains of life in uniprotkb. **(b)** Quality assurance and control (QA/QC) preprocessing steps. **(c)** The homology reduction framework designed to minimize information leakage. **(d)** Model training, validation, testing and associated evaluation experiments.

We propose a novel comprehensive dataset (*SCL2205*) for application in SCL modelling, unlike the minimally described benchmarks used in most SCL models. For reproducibility, we discuss in detail the processes and design choices used to generate the dataset. This work establishes a new benchmark dataset that will empower researchers to build more sustainable, robust, and trustworthy tools for the next frontier of AI in spatial genomic discovery. Fig. 1 is a graphical summary of the study.

## 2 Materials and methods 

### 2.1 Terminology

#### 2.1.1 Sequence homology

According to Medical Subject Headings (MeSH), it is the degree of similarity between sequences. The similarity is attributed to descent from a common ancestor and can be based on percentage sequence identity and/or percentage positive substitutions. However, we acknowledge that an alternative definition for homology exists (Higdon *et al.* 2010). The MeSH definition is assumed throughout this study, which is consistent with its usage in the SCL modelling space. While homology within diverse sets is important in modelling, over-representation due to excess sequence relatedness can be problematic. Mitigation is needed to avoid over-weighting closely related sequences. In database parlance, sequence homology is commonly synonymous with *redundancy*; therefore, redundancy reduction is associated with homology mitigation.

#### 2.1.2 Homology reduction

It is the process of minimizing sequence homology across or within datasets. In this study, we distinguish *across/inter* and *within/intra* dataset homology reduction as *overlap* and *redundancy*, respectively.

#### 2.1.3 Data leakage

Refers to data overlap across independent datasets. When in excess, it is undesirable in model training, where sufficient separation is needed across the *training-validation* and *training-testing* sets. Separation serves two major purposes: (i) enabling accurate model performance assessment and (ii) ensuring model inferential robustness to unseen data.

#### 2.1.4 Data augmentation

Data augmentation increases the size and/or enriches the quality of available data to enhance model performance, without explicitly collecting additional samples. It helps mitigate the problem of minimal training data, especially for supervised learning. The details of data augmentation are elaborately covered elsewhere (Shorten and Khoshgoftaar 2019, Feng *et al.* 2021, Shorten *et al.* 2021). From a broad perspective, it may be categorized into three approaches: (i) input-level augmentation, which involves transformations applied in the data space; (ii) embedding-level augmentation, which operates in the feature space and targets intrinsic relationships and semantics; and (iii) hybrid augmentation, which combines elements of both input- and embedding-level strategies. Alternatively, from the perspective of the augmentation source, methods may be classified as either *internal* or *external*. Internal augmentation entails directly modifying the existing data, e.g. reordering elements within a sequence. In contrast, external augmentation involves incorporating additional information, such as generating sequence profiles through sequence alignments. Although protein SCL prediction borrows substantially from the text domain of sequential learning, it has unique requirements and/or characteristics. Therefore, most of the natural language processing (NLP) augmentation techniques are not (yet) implemented in the SCL modelling domain. However, since the latter is affected by imbalanced and limited labelled data, other augmentation techniques are applied, mainly homology augmentation, yet not without inherent limitations, especially since it counteracts homology reduction. Homology augmentation can be categorized as embedding augmentation, whereas label mapping falls under input augmentation.

#### 2.1.5 Homology augmentation

We define homology augmentation as sequence data enhancements that involve database searches to retrieve additional sequences related to the original dataset. The additional sequences can be directly included in the original set or used to build sequence profiles from multiple sequence alignment (MSA). The latter is common with DL SCL predictors.

### 2.2 Data source

#### 2.2.1 Ethical considerations

No ethical pre-approval was required. The raw data were retrieved from a public protein sequence database, UniProtKB, which does not contain any personal information.

#### 2.2.2 The universal protein knowledgebase

The UniProtKB is the central repository for aggregating functional information on proteins characterized by consistent, accurate, and rich annotations. UniProtKB annotations include universally accepted cross-references, ontologies, classifications, and quality scores informed by experimental and computational evidence (UniProtConsortium *et al*. 2023). The knowledge base consists of two sections: (i) UniProtKB/Swiss-Prot for manually annotated records based on literature information and expert-evaluated computational analysis and (ii) UniProtKB/TrEMBL for computationally analysed records awaiting full manual curation (UniProtConsortium *et al*. 2023). We used the former source because of the quality assurance of its records. More than 95% of proteins in UniProtKB are derived from translations of coding sequences (CDS) submitted to the International Nucleotide Sequence Database Collaboration (INSDC) databases (UniProtConsortium *et al.* 2023). In UniProtKB/Swiss-Prot, all protein products encoded by a gene in a given species are represented in a single record; i.e. identical sequences are captured as a single record. However, if the sequences are from different species, they form different records (UniProtConsortium *et al.* 2023).

#### 2.2.3 Protein subcellular location labels

Most SCL predictors are trained with *supervision*. The *Subcellular location* tab of UniProtKB contains the protein cellular location annotations, which are used as labels to supervise training. The UniProtKB provides location and topology information for the mature protein in the cell using a structured hierarchy of controlled vocabulary, except for the *Note*, which is free text (UniProtConsortium *et al*. 2023). Because proteins are of diverse origins such as mitochondrial, plastid or nuclear , it is important to note that the labels represent the mature protein, i.e. fully processed and functional, resulting from post-translation modification (PTM) and proteolytic processing. It is the ultimate version associated with a biological function. Therefore, filtering sequences on genomic origin (mitochondria/plastid/nuclear) (Armenteros *et al.* 2017, Thumuluri *et al.* 2022) may be unnecessary in general predictor training.

#### 2.2.4 Raw data

Protein sequences and their annotation information were manually retrieved in tabular format from the latest release of the *Reviewed* subset of UniProtKB (UniProtKB/Swiss-Prot, Release 2022_05; date: 24 January 2023). Each record includes a stable and unique *Entry* identifier, which is essential for provenance and reproducibility.

### 2.3 Data inclusion criteria

A total of 469 935 sequence records were downloaded. The following five filtering processes were applied; *data field name: process description*:

- *Subcellular location [CC]*: records lacking SCL annotation removed.
- *Subcellular location [CC]*: annotations with an evidence code ontology (ECO) code of *ECO:0000269* for the evidence were retained—experimentally determined. These annotations are a credible basis for supervisory learning.
- *Taxonomic lineage*: retained records belonging to the eukaryotic taxonomic group using *Eukaryota (super-kingdom)* as the match phrase in the description. The eukaryotic cell is very similar across species; therefore, we minimize data fragmentation across taxonomic *Kingdoms* by aggregating at the super-kingdom level.
- *Annotation*: annotations with a quality score of at least three were retained, being the median and the discrete score above the midpoint. Although often overlooked, the cut-off enforces stringent reference-data quality to minimize compounding errors.
- *Length*: sequences with at least 30 and at most 5000 amino acids. It has been shown that biological function is preserved in shorter sequences (Fan and Wang 2003); however, most SCL predictors adopt a minimum sequence length of 30–40 amino acids.

The dataset used in most SCL predictors incorporates minimal preprocessing before proceeding to the homology reduction step. However, we harness more from the data than in previous research. The DeepLoc dataset (Armenteros *et al.* 2017, Thumuluri *et al.* 2022) incorporates label mapping for various locations. However, it is minimal, based on an older database release and may contain some avoidable noise. For instance, while organellar-membrane-localizing proteins are mapped to their respective organelles, membranes, relative to the organelles, are structurally and functionally related across organelles, in a general sense.

Therefore, it might be better to consider them as a location entity, at least for a general SCL predictor, rather than organelle-specific. The latter approach is adopted in some membrane protein predictors, when predicting endomembrane system and secretory pathway proteins (Kaleel *et al.* 2020) or membrane and non-membrane proteins (Kaleel *et al.* 2021).

#### 2.3.1 Label mapping

We exploited the SCL database, controlled vocabulary definitions, and established spatial cellular organization (Mercier *et al.* 2022, UniProtConsortium *et al*. 2023) to map infrequently occurring SCL labels to a predefined set of prediction targets.

The process began by extracting and reorganizing the label information from the structured annotation text using custom code (see ‘Data and code availability’ section for availability). The result is a list of concise, sorted, and unique SCL label(s) for each sequence. Subsequently, label frequencies were calculated and ranked in descending order. Afterwards, manual label mapping was performed according to the following criteria, either as combinations or in isolation:

- Harmonization of commonly interchangeable localization terms, particularly *Cytosol*↔*Cytoplasm* and *Extracellular*↔  *Secreted* pairs.
- Mapping of specific localizations to broader familial categories where appropriate, particularly for plastid-related annotations.
- Assignment of sub-compartment membrane annotations to the membrane category, reflecting the shared membrane-associated localization.
- Mapping of localizations within a broader cellular compartment to the corresponding parent compartment (e.g. nuclear sub-compartments to nucleus), except for *Cytoplasm* and *Secreted* categories.
- Consultation of gene ontology (GO) ancestor relationships (the *Ancestor Chart*) to guide mappings when the preceding criteria were insufficient.

To minimize the risk of subjective mapping, Criteria iv and v were applied sparingly and only after consideration of Criteria i–iii. Priority was given to initially observed labels of higher frequency. Mapping was not exhaustive, especially for multi-label combinations, because mapping complexity increases with the number of constituent labels. Consequently, the level of localization granularity was determined *a priori* according to the intended prediction targets and the availability of sufficient reference sequences for model development.

**Figure 2:**
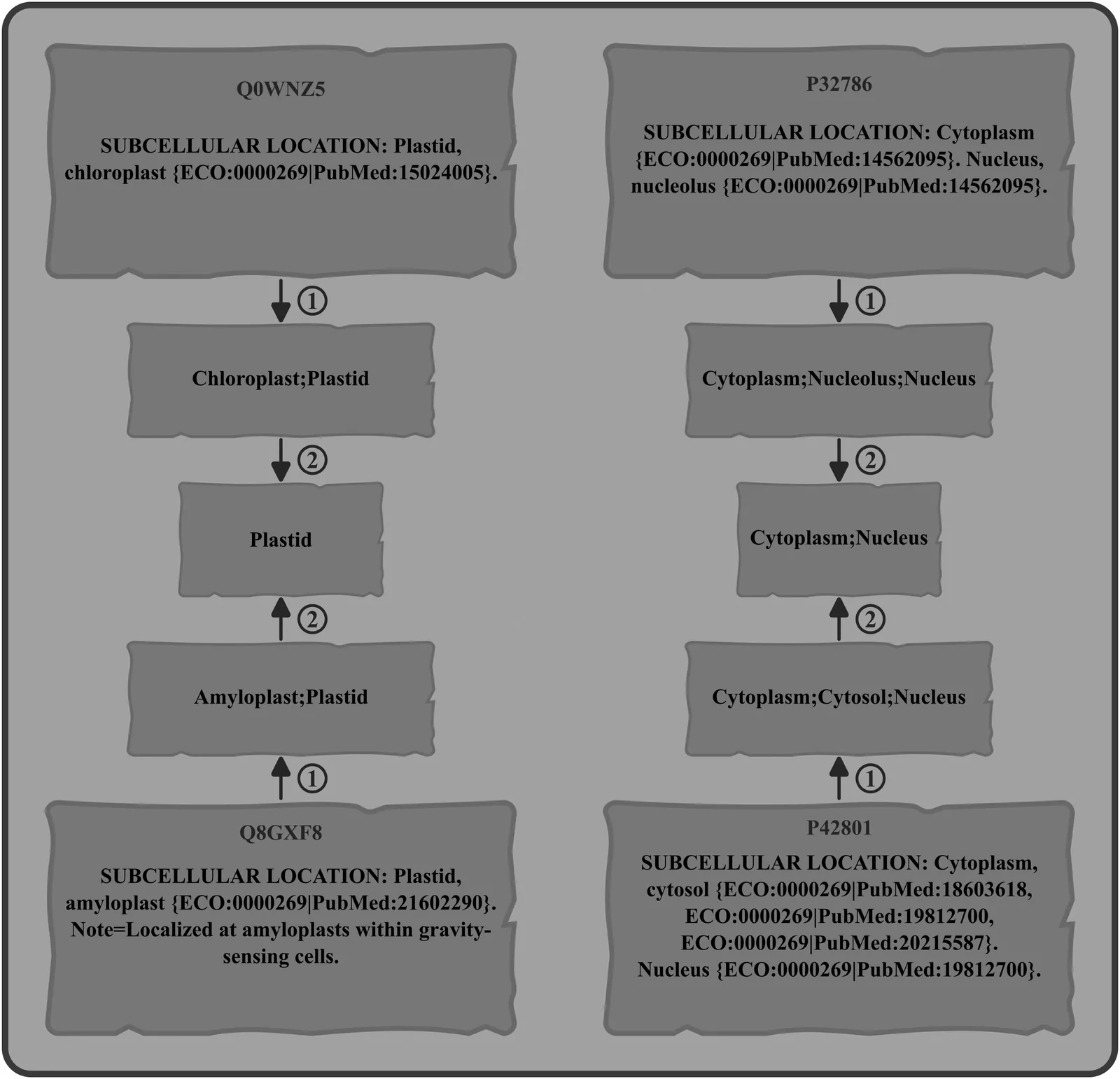
Illustrating SCL label mapping using four proteins. The left and right panels represent *single-label* and *multi-label* protein mapping, respectively. **Step 1**: programmatically extract labels from structured annotation text using custom code (see ‘Data and code availability’ section for availability). **Step 2**: manually map sub-location (sub-compartment) to location labels and harmonize interchangeable labels.

For completeness, *single-label* and *multi-label* proteins were included in the final dataset (*SCL2205*). However, for simplicity, only the *single-label* proteins were used for the experiments. Fig. 2 illustrates the process of label mapping. Ultimately, the process resulted in a substantial increase in sequence count (see Table 1), which is valuable for training.

**Table 1:** A summary of the SL composition showing the mapping effect.^a^

Label	Original	Mapped	Fold increase
Membrane	290	5616	19.37
Nucleus	3621	4721	1.30
Secreted	3067	3357	1.09
Cytoplasm	2209	2706	1.22
Cytoplasm; nucleus^b^	2167	2618	1.21
Mitochondrion	838	1062	1.27
Plastid	6	622	103.67
Cent; cytop; cytos; MTOC^b^	92	338	3.67
Cytoplasm; membrane^b^	88	284	3.23
Cytoplasm; cytoskeleton^b^	244	270	1.11
ER	150	204	1.36
Cell projection	6	194	32.33
Peroxisome	144	160	1.11
**Totals**	**12 922**	**22 152**	**1.71**

a The first and second columns are the *before* and *after* protein counts, respectively. The fold increase is captured in the last column. Overall, the sample size grew by 71%. When considering only the single-label proteins, the numbers improved by 80%. For brevity, *centrosome; cytoplasm; cytoskeleton; microtubule organizing center* is shortened as *Cent; Cytop; Cytos; MTOC*. The total fold-increase (1.71) represents the gains between umapped (12 922) and mapped (22 152) labels.

b Multiple-localization.

#### 2.3.2 Homology reduction

Essentially, homology reduction is common in protein modelling for two reasons: to mitigate data leakage and avoid learning bias; it mainly relies on sequence alignment. The *CD-HIT* tool (Fu *et al.* 2012), which is based on position-specific iterative BLAST (PSI-BLAST), is widely used by DL predictors to facilitate homology reduction. *CD-HIT* prefers longer sequences, despite short and long sequences having functional roles. However, at least two issues arise: unnatural input data distribution and loss of functional domain (pattern) representation that could be useful for learning SCL. Consequently, we implemented a custom variant sequence similarity algorithm, which is still based on local alignment sequence tool (BLAST), factors sequence-pair length, but without preference for longer sequences, and conveniently operates on *training-testing* split dataset models for ML; avoiding multi-stage homology reduction regardless of the homology threshold. The sequence similarity algorithm is detailed in the *Methods* section of File 1, available as supplementary data at *Bioinformatics* online.

**Figure 3:**
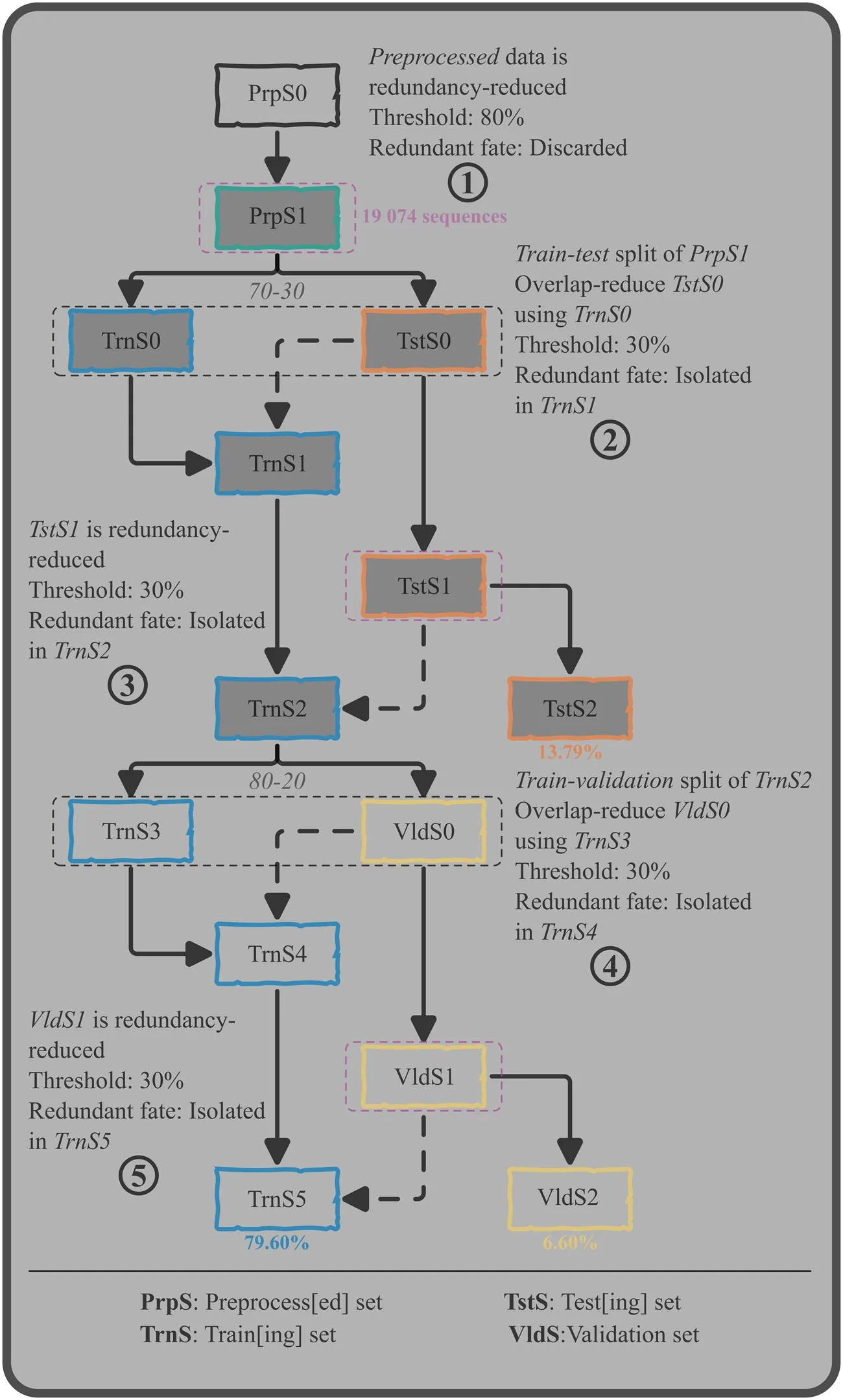
Summary illustration of the three-step homology reduction process based on global pairwise alignments. Solid-line arrows indicate data processing flows, broken-line arrows represent data isolation, and bifurcating arrows represent data splits (split ratios are included at the nodes). Magenta, dashed rectangles denote redundancy reduction, while the black ones represent overlap reduction. The shaded rectangles highlight the repeating *motif* within the homology reduction pipeline. The process takes the 22 152 preprocessed (*PrpS0*) instances as input. It then begins with a global redundancy reduction that results in 19 074 instances (*PrpS1*; 100%), resulting in 15 183 instances in the training set (*TrnS5*; 79.60%), 1260 in the validation (*aka* discovery or development) set (*VldS2*; 6.60%), and 2631 as the held-out testing set (*TstS2*; 13.79%).

Based on the reasons already provided, we had the following goals for our homology reduction pipeline (Fig. 3):

- **Mitigating data leakage—**similar samples stay in a bin; *training* or *testing*, not both.
- **Avoiding learning bias; imbalance, by implication—**maintain a particular sequence similarity threshold within a bin.

We then proceeded with a three-step homology reduction strategy based on similarity thresholds and dataset partitions. In the first step, we minimize overweighting associated with imbalanced classes and high sequence homology during training by using an 80% sequence similarity threshold to redundancy-reduce the preprocessed dataset (Fig. 3, *PrpS0*). The second and third steps use a 30% sequence similarity threshold on the partitioned data. The 30% threshold is based on a convention in most SCL predictors, supported by studies suggesting a *twilight* homology-range for functional inference (transfer) (Rost 1999, Nair and Rost 2002). The testing set (*TstS0*; subject; 30%) was overlap-reduced against the training set (*TrnS0*; query; 70%) and the testing set (subject and query) redundancy-reduced, respectively. Whereas the second step mitigates data leakage, the third step mitigates evaluation bias, which is associated with imbalanced classes and sample relatedness. Sequences that overlapped or were redundant, relative to the testing set, were isolated (binned; Fig. 3 broken arrows) in the training set to align with the goals.

Moreover, we used the resulting training set to generate two dataset tracks for model training, based on a common model development data partitioning approach: *training*, *validation*, and *testing*; and *cross-validation* and *testing*. Henceforth, we refer to the two tracks as *training-validation-testing* (TVT) and *cross-validation-testing* (CVT), respectively, and collectively designate them *SCL2205*. Note that the held-out testing set is shared because the original dataset is the same. Therefore, henceforth we refer to the partitions associated with the model training process as *training-validation* (TV) and *cross-validation* (CV). As before, TV and CV sets were subjected to the second and third steps of homology reduction. This was performed for all five folds for the CV set. In the TV set, the *training* (Fig. 3, *TrnS3*) and *validation* (Fig. 3, *ValS0*) sets were split at 80% and 20%, respectively.

**Note.** To ensure reproducibility, all data partitioning was performed using the train_test_split function from the *scikit-learn* library, with a consistent random seed of 2023 maintained throughout the study. The random seeds for the 10 bootstrap samples used to quantify data leakage ranged from 2023−2032. Other software details are in File 1—under *Software and tools information*, available as supplementary data at *Bioinformatics* online.

## 3 Experiments

### 3.1 Manual mapping impact

We aimed to assess the contribution of manual label mapping to model generalization, compared to using the original labels. Using $\mathrm{SCL}2205_{s}$ as reference, we generated $\mathrm{SCL}2205_{s}^{\mathrm{nomap}}$ by copying $\mathrm{SCL}2205_{s}$ and removing all sequences associated with the label-mapping augmentation process. Since very few samples in the *Cell projection* and *Plastid* categories remained when considering the original labelling, we omitted them from the modelling process. The corresponding partitions of the sets were used to train two convolutional neural network (CNN)-based networks (see Section 3.3.2). The independent test sets were redundancy-reduced, relative to the two datasets and used for evaluation.

### 3.2 Quantifying data leakage from homologous augmentation

In ML learning, including SCL modelling, a degree of relatedness in the datasets is necessary for learning, testing, and inference. However, care must be taken to minimize information leakage that could hamper accurate performance evaluation or reduce real-world model utility, especially in imbalanced-class classification settings. To analogize, while a student is examined on concepts taught in class, they should not be examined on the exact examples encountered in class; rather, assessment should evaluate their ability to generalize those concepts to unseen problems. To explore the potential for information leakage arising from homology augmentation, we posed the following question.

*How does homology augmentation contribute to and impact training-testing dataset overlap, especially for predictors based on pre-model-training homology reduction?*

To address the question, we designed an experiment using the TVT dataset. Whilst maintaining class proportions, we randomly drew, with replacement, 10 samples of 10% (n=1280; *random-training-seqs-A*) sequences of the training set (Fig. 3; *TrnS5* minus multi-localizing sequences; 12 807) sequences and queried the *RefSeq* (Version 5 of 202410260537) database for homologues using the conventional blast global alignment. Our psi-blast parameters included: evalue = 0.001; num_iterations = 3; matrix = BLOSUM62, word_size = 3 and max_target_seqs = 500. We retrieved the full set of homologous sequences (designated *HmgS1*) using their parsed sequence accessions per sample, followed by overlap reduction (using the 30% threshold applied during the initial homology reduction process) on *combined validation and testing set (CVTS)*; using the respective *HmgS1* instances as a query. Subsequently, we used the proportion of CVTS sequences overlapping the respective *HmgS1* instances as a proxy for the extent of overlap. That is, dividing the number of overlapping sequences by the total number of sequences within CVTS. The rationale is that using overlapping sequences to generate profiles propagates the overlap (leakage) to the resulting sequence encoding.

#### 3.2.1 Ablation of augmenting sequences from homology reduction in leakage calculation

To nullify the contribution of the overlapping sequences isolated within the training set to leakage calculation, we created *random-training-seqs-B* instances by removing the augmenting sequences resulting from the homology reduction process (shown as broken arrows in Fig. 3) from the corresponding *random-training-seqs-A* instances. We then filtered out all hits associated with the removed sequences from their respective *HmgS1* sets. Subsequently, we used 10 sets of overlap reduction for the test set using the remaining portions (designated *HmgS2* instances). This enabled us to observe the impact of these specific sequences on the extent of overlap, i.e. to determine ‘net leakage’.

### 3.3 Dataset benchmarking

To benchmark the reliability of our newly developed SCL dataset (*SCL2205*; *n* = 19 074), we assessed its performance against a comparable state-of-the-art (SoTA): DeepLoc2, redundancy-reduced at an 80% threshold [SwissProt train-validation (*DEEP-TV*); *n* = 22 126]. Finally, we evaluated these using two independent sets: (*DEEP-SS*) and (*DEEP-HPA*) (Thumuluri *et al.* 2022).

#### 3.3.1 Dataset pre-processing

*SCL2205* and *DEEP-TV* were redundancy-reduced similarly. For training, *SCL2205* and *DEEP-TV* were split into *training* (*n *= 10 680; 12 390 [56%]), *validation* (*n *= 2671; 3098 [14%]) and *testing* (*n *= 5723; 6638 [30%]) partitions, respectively. The splits were randomized and stratified by labels. All *DEEP-TV* sequences localizing in the *Golgi apparatus* were excluded because they were fewer than n=100, which was the minimum cut-off used with *SCL2205*. For brevity, experiments were performed using single-label classes, which were intuitively coded $\mathrm{SCL}2205_{s}$ and $\mathrm{DEEP}-TV_{s}$, respectively. See (Table 2) for the class distribution between datasets and partitions.

**Table 2:** Label-wise distribution across datasets and partitions (train [Trn], validation [Vld], and testing [Tst]) for single-label labels only.^a^

Label	$\mathrm{SCL}2205_{s}$			$\mathrm{DEEP}-TV_{s}$		
	Trn	Vld	Tst	Trn	Vld	Tst
MEM	2642	661	1415	1117	599	279
NUC	2340	585	1254	2581	1383	646
SEC	1475	369	791	1307	701	327
CYT	1380	345	740	2073	1111	518
MIT	530	132	284	617	331	154
PLA	329	82	176	332	178	83
ER	104	26	55	131	70	33
CEP^b^	98	25	53	NA	NA	NA
PER	76	19	41	75	40	19
LYS/VAC^b^	NA	NA	NA	106	57	26
**Totals**	**8974**	**2244**	**4809**	**8339**	**4470**	**2085**
**Coverage**	**56%**	**14%**	**30%**	**56%**	**14%**	**30%**

a There is a notable difference for the *Membrane* class. *DEEP-TV* has a higher number of training examples than *SCL2205* in 60% of the classes. The totals correspond to the number of samples (sequences) across the different data partitions per column, while the coverage is their respective percentage proportions. For brevity: membrane (MEM), nucleus (NUC), secreted (SEC), cytoplasm (CYT), mitochondrion (MIT), plastid (PLA), endoplasmic reticulum (ER), cell projection (CEP), peroxisome (PER), and lysosome/vacuole (LYS/VAC).

b Missing for the corresponding dataset and omitted during dataset-comparison model training.

Since the *DEEP-SS* independent dataset was initially labelled by sorting signals, it was therefore relabelled. We retrieved subcellular location labels from UniProtKB using persistent sequence accessions. Only sequences with matching UniProtKB accessions were considered. The human protein atlas (HPA) dataset labels were similar to our training data.

#### 3.3.2 Test modelling

##### 3.3.2.1 Input encoding

For the embedding CNN, SentencePiece (Kudo 2018) tokenizers were trained on the respective training sequences and subsequently used to generate tokenized inputs for the models. The PLM used its own pre-trained tokenizer. Further configuration details are provided in File 1—*Model architectures*, available as supplementary data at *Bioinformatics* online.

##### 3.3.2.2 Architecture

Briefly, the CNN embedding architecture comprised of four 1D convolutional layers with ReLU activation, organized into two stacks. Three parallel input layers were used and their outputs merged in the fourth layer, which additionally incorporated dropout regularization. For PLM embedding, we used an independent pre-trained PLM—Rostlab/prot_t5_xl_uniref50 (*Prot5*)—by the Rost Lab (Elnaggar *et al.* 2022), using the *Transformers* api (Wolf *et al.* 2020) from *Hugging Face*. *Prot5* is a leading SoTA PLM and has consistently demonstrated strong performance across SCL predictors (Stärk *et al.* 2021, Thumuluri *et al.* 2022). To ensure comparability between embedding strategies, latent representations generated by both the CNN and PLM models were mapped to prediction targets using the same fully connected neural network (FCNN), implemented as a multi-layer perceptron (MLP) with two hidden layers. Complete architectural details are provided in File 1—*Model architectures*, available as supplementary data at *Bioinformatics* online.

##### 3.3.2.3 Training

Training continued until the model overfitted. That is, a sustained increase in (training-validation) loss after an initial decrease, as observed in the loss plot. For computational robustness (training was done on a managed GPU computing cluster), each model training phase was run three times, followed by weight averaging to instantiate the final model used for testing. The model snapshot with validation accuracy peaking before a sustained rise of the training loss was saved for each phase.

##### 3.3.2.4 Evaluation

Evaluations based on independent test sets were compared using multiple strategies, either in isolation or as combinations: *macro* and *per-*class area under the precision–recall curve (PR-AUC) metrics for ranking and positive retrievals, McNemar’s error rate test, and stratified bootstrapping on metric differences for uncertainty quantification. Evaluation testing was performed using independent test sets *DEEP-SS* and *DEEP-HPA*, see Table 3.

**Table 3:** Label-wise counts for independent test datasets.

Label	*DEEP-SS*	*DEEP-HPA*
Secreted^a^	522	NA
Membrane	170	161
Mitochondrion	111	175
Peroxisome	77	7
Nucleus	63	664
Cytoplasm	10	263
ER	5	31
Plastid^a^	1	NA
**Totals**	**958**	**1301**

a Footnote a: Per-class sample counts for the two independent test datasets, corresponding to the eight classes shared with the training sets. The total number of samples in each dataset is shown in bold.

Footnote b: Missing for the corresponding dataset.

#### 3.3.3 $\mathrm{SCL}2205_{s}$  *versus* $\mathrm{DEEP}-TV_{s}$  *comparison*

To compare datasets as holistic stand-alone entities, we used the corresponding partitions of $\mathrm{SCL}2205_{s}$ and $\mathrm{DEEP}-TV_{s}$ to train an *ad hoc* CNN-based model; we also isolated model effects by training an independent plm-based model. The models resulting from training with $\mathrm{SCL}2205_{s}$ and *DEEP-TV_s_*; for brevity, *Model A* and *Model B*, respectively, were then evaluated. The independent test datasets were overlap-reduced with each corresponding *training* set (see post-ablation class distributions in Table 3, available as supplementary data at *Bioinformatics* online).

### 3.4 DEEP-SS and (DEEP-HPA) independent test sets

We highlight distinct characteristics of these two external evaluation sets. *DEEP-SS* is drawn from universal protein (UniProt)-SwissProt, the source of training data for both sets; therefore, it can be considered an *in-distribution* set. In contrast, (*DEEP-HPA*) is based on SCL annotations from the HPA, which uses a different annotation strategy. SCL in HPA is diversely investigated; using isolated or integrated techniques comprising of immunocytochemistry-immunofluorescence (ICC-IF), confocal microscopy, and staining (Thul *et al.* 2017). Moreover, the distinction is highlighted by the extent of overlap with the *SCL2205* and *DEEP-TV* training sets, as captured in various overlap-reduction instances, see Tables 2 and 3, available as supplementary data at *Bioinformatics* online. Furthermore, while the uniprot-SwissProt data covers multiple taxa, HPA covers only humans. Therefore, we consider (*DEEP-HPA*) out-of-distribution (OOD).

## 4 Results

### 4.1 Mapping improved generalization

**Figure 4:**
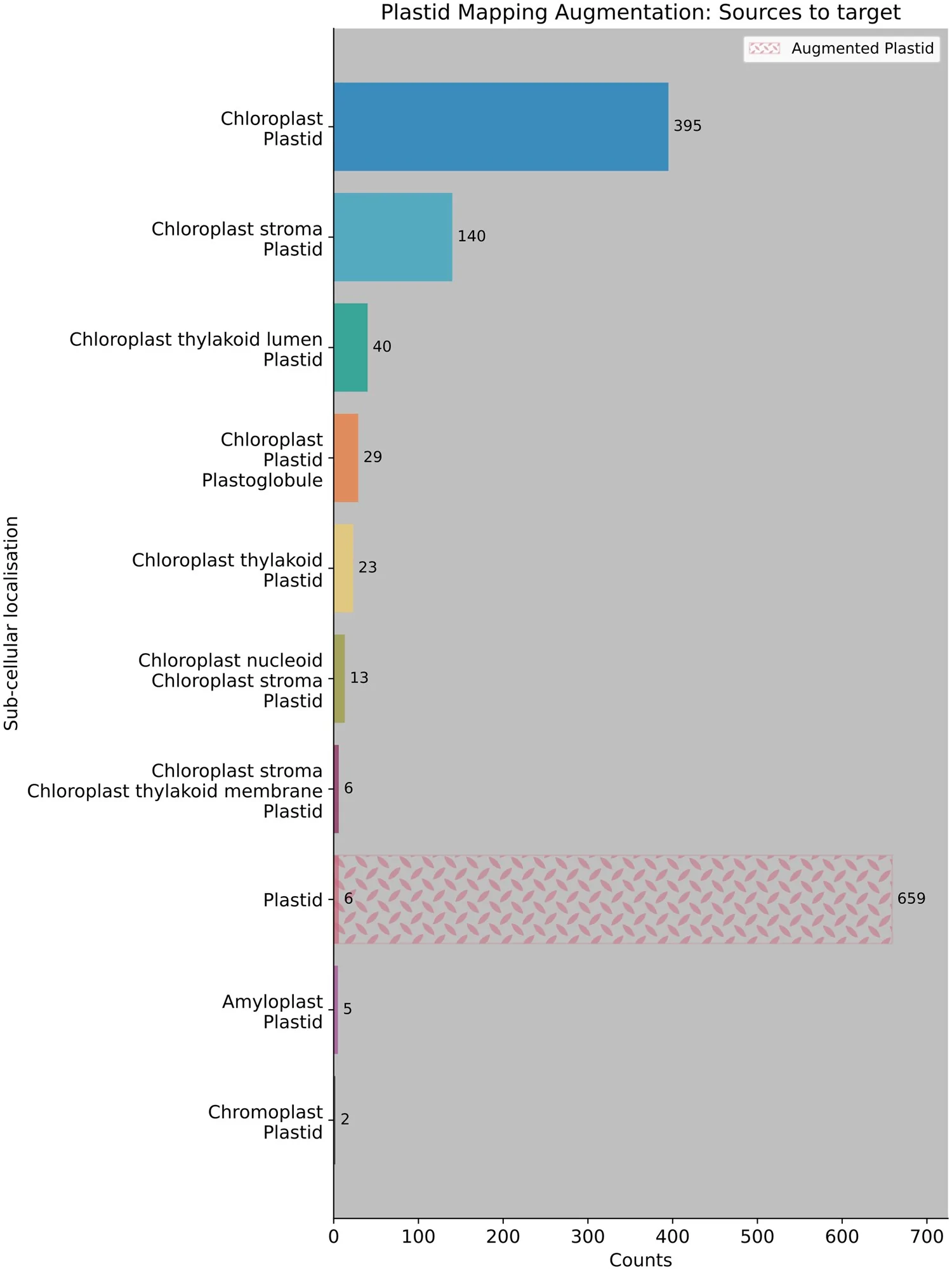
Label-mapping frequencies for plastid augmentation involving nine locations. We reported the highest fold increase for plastid (103.67-fold).

We exploited manual label mapping to increase target diversity and boost the number of training samples, to improve model generalizability and performance. A representative outcome of the mapping process using *Plastid—*the most impacted location—is illustrated in Fig. 4. For clarity, we refer to the model using label mapping as *Model A* and the model using native labelling as *Model B*.

We observed a substantial improvement in the macro PR-AUC for *DEEP-SS* when using label mapping (an increase of 9.0% points); however, the improvement for *DEEP-HPA* was marginal (1.5% points). Full details, including per-class PR-AUC with their respective prevalences, are provided in Table 4, available as supplementary data at *Bioinformatics* online. Overall, label mapping improved model performance across most classes; however, we observed marginal deterioration in the *Cytoplasm* and *Secreted* categories for *DEEP-SS* and *Nucleus* for *DEEP-HPA*.

**Table 4:** Leakage summary statistics across the 10 bootstrap replicates.^a^

S/No.	RTS-B	*HmgS1*	*HmgS2*	Overlaps
1	896	665 860	4,79 011	155
2	920	651 670	480 965	159
3	859	652 505	453 060	163
4	896	651 567	465 961	150
5	885	656 845	468 566	178
6	880	652 782	451 776	175
7	895	659 948	474 071	181
8	890	650 062	463 815	161
9	905	657 215	479 291	166
10	891	653 894	462 365	173
**Avg.**	**892**	**655 235**	**467 888**	**166**
**Std. dev.**	**15.9**	**4828.2**	**10507.8**	**10.3**

a The columns are: replicate sample enumeration, de-augmented replicate (*random-training-seqs-B*), homologous set 1, homologous set 2, and the number of samples overlapping the *cvts* set, respectively. At the bottom of the table are the averages (Avg.) for each column and the corresponding standard deviations (Std. dev.), shown in bold.

An asymptotic McNemar’s test with continuity correction revealed a significant performance difference between *Model A* and *Model B* on both test sets. This test evaluates error rates at a fixed threshold. For *DEEP-SS*, $\chi^{2}(df=1)=8.2,\;p<0.004$, with *Model A* outperforming *Model B* [*b: A* correct, *B* wrong = 108; *c: A* wrong, *B* correct = 69; *Effect Size* Z (standardized discordance statistic) = 2.9]. In contrast, for *DEEP-HPA*, $\chi^{2}(df=1)=278.8,\;p<0.001$, with *Model B* outperforming *Model A* (b=610, c=149; *Effect Size* Z=−16.7).

**Figure 5:**
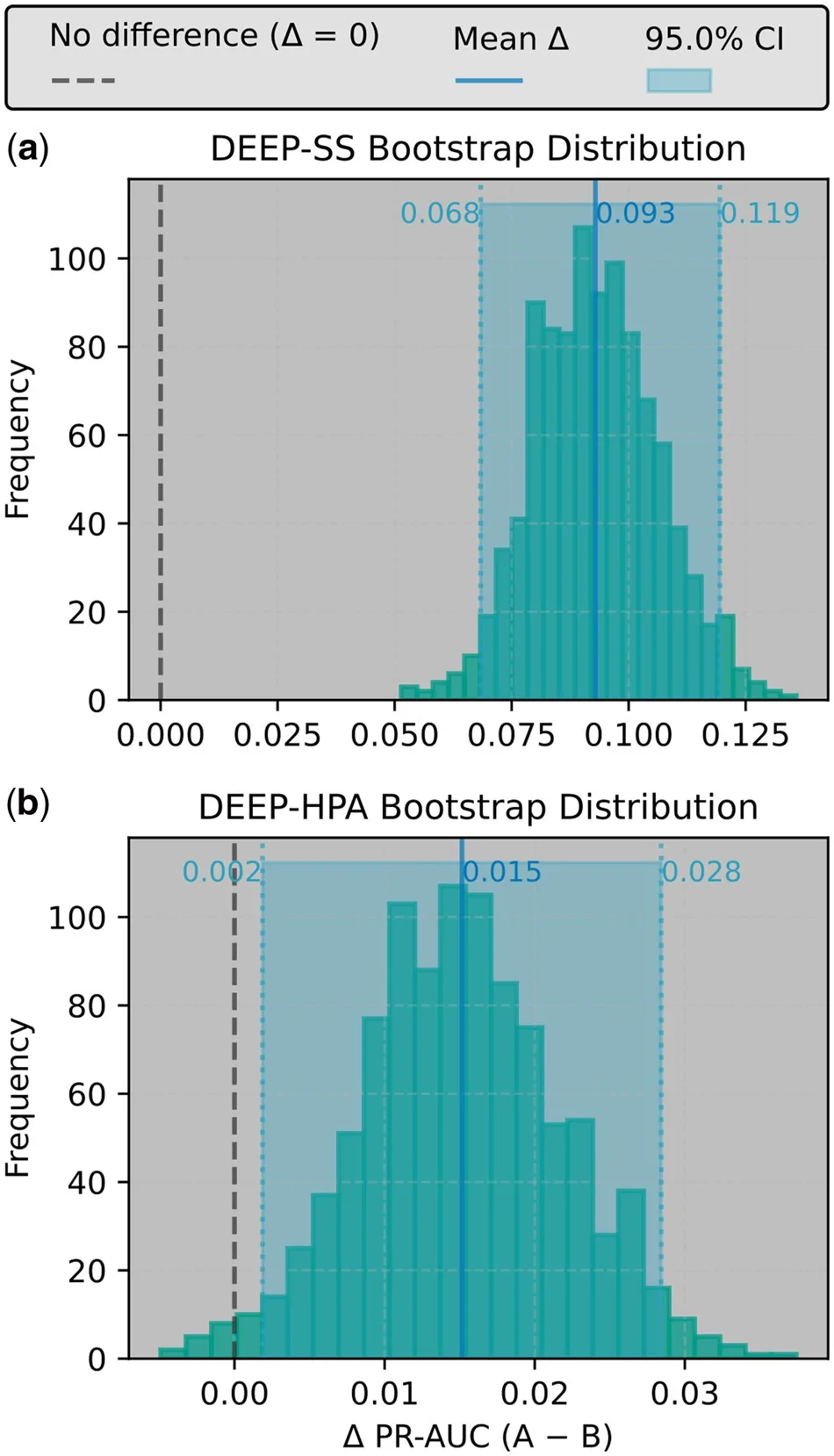
Histogram of the distribution of 1000 bootstrap PR-AUC metric differences between models *A* and *B* as trained on *SCL2205*${\;}_{s}^{\mathrm{map}}$ and *SCL2205*${\;}_{s}^{\mathrm{nomap}}$, respectively. The top panel **(a)** was tested on *DEEP-SS* (A>B), whereas the bottom panel **(b)** was tested on (*DEEP-HPA*) (A>B).

The significant performance difference between *Model A* and *Model B* was supported by the stratified paired bootstrap confidence interval (CI) test. However, although *Model A* still, as in McNemar’s test, outperformed *Model B* on the *DEEP-SS* set, the reverse was observed for the *DEEP-HPA* set in this test, where the label mapping of *Model A* proved superior. Fig. 5 illustrates the uncertainty levels based on the macro PR-AUC differences between the two models.

### 4.2 Data leakage from homology augmentation

We show and quantify that homology augmentation can partially undo prior *training-testing* dataset homology reduction. Interestingly, we found that on average, 10% of the training set, for homology augmentation, resulted in 5.2% overlap in the *training-testing* sequences.

The average number of sequences removed from *random-training-seqs-A* was 388, while a corresponding 892 remained (*random-training-seqs-B*), which corresponds to 10% of the training set (the single-location subset of *TrnS5* with all augmenting sequences removed). Averagely, *HmgS1* had 655 235 sequences, while *HmgS2* had 467 888 a 28.6% (*n* = 187 347) reduction in the initial homology-augmenting sequence hits. The detailed description across the 10 samples is provided in Table 4. These results underscore the need for care when homology enhancement is performed on training data. If ignored, consequences may include inflated performance metrics and hampered generalization to unseen real-world data. Additionally, the findings provide a baseline quantification, arguably the first such indication, for the amount of overlap resulting from database search augmentation, on the same sequence similarity measurement scale.

### 4.3 A robust benchmark dataset for subcellular localization prediction

We developed *SCL2205* from the latest UniProtKB release, with extensive quality preprocessing to ensure reliability, manual-mapping augmentation to increase training data and improve generalization, and stringent data partitioning to minimize data leakage. Whereas we observed a 1.71-fold increase in data during the development process, the inherent imbalance across data classes persists, albeit reduced. Due to the imbalance, we evaluated the dataset’s performance using the macro PR-AUC metric. The precision–recall curve (PRC) curve plots the trade-off between recall [sensitivity/true positive rate (TPR)] and precision [specificity/positive predictive value (PPV)] across multiple thresholds. On the other hand, the PR-AUC score summarizes the PRC curve as a number. Unlike area under the ROC curve (ROC-AUC), where the binary baseline is 0.5, positive-class prevalence is the baseline in PR-AUC.

Next, we consider the results for the two model architectures used for dataset comparisons. Table 5 provides the global PR-AUC scores, while the per-class scores are summarized in Tables 5 and 6, available as supplementary data at *Bioinformatics* online.

**Table 5:** *SCL2205*_*s*_ and *DEEP-TV*_*s*_ performance comparisons: a summary of the macro PR-AUC across model variants and test datasets.^a^

Dataset and architecture	Baseline prevalence	*SCL2205*_*s*_ 8-class	*DEEP-TV_s_* 8-class
***DEEP-SS*_CNN_**	0.143^b^	0.257	0.334
***DEEP-HPA*_CNN_**	0.167^b^	0.194	0.184
***DEEP-SS*_PLM_**	0.143^b^	0.561	0.453
***DEEP-HPA*_PLM_**	0.167^b^	0.345	0.359

a Despite using vanilla models, all scores exceeded the expected random guessing level. We observed the most significant gains in the PLM-based model—up to 10.8% points in favour of *SCL2205*_*s*_.

b We computed the average prevalence across classes for each test set.

**Figure 6:**
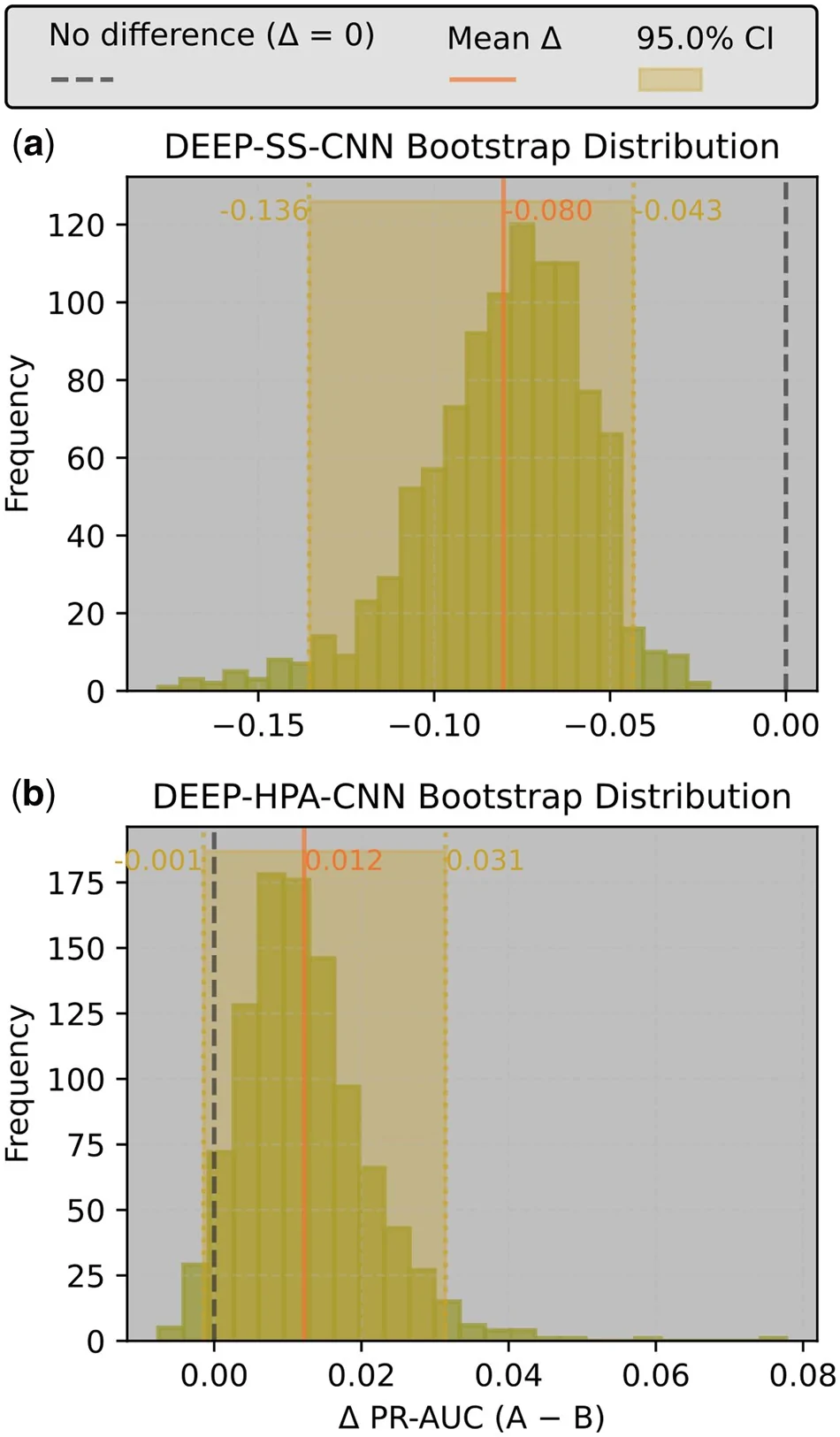
Histogram of the distribution of 1000 bootstrap PR-AUC metric differences between CNN-based models *A* and *B* as trained on *SCL2205*${\;}_{s}$ and *DEEP-TV*${\;}_{s}$, respectively. The top panel **(a)** was tested on *DEEP-SS* (B>A), whereas the bottom panel **(b)** was tested on (*DEEP-HPA*) (no significant difference).

*CNN-based architecture* An asymptotic McNemar’s test with continuity correction showed a significant difference between the models trained on *SCL2205*${\;}_{s}$ (*Model A*) and *DEEP-TV*${\;}_{s}$ (*Model B*) when evaluated on the two test sets. For *DEEP-SS*, $\chi^{2}(df=1)=18.1,\;p<0.001;\;(B>A\;[b=90,c=158])$ with an Effect Size Z=−4.3. *DEEP-HPA*: $\chi^{2}(df=1)=143.0,\;p<0.001;\;(A>B\;[b=145,c=0])$ with an Effect Size Z=12.0. We observed a similar pattern using the stratified bootstrap on the PR-AUC metric difference test (Δ=A−B). For *DEEP-SS*, $\bar{x}=-0.080,\;p<0.001;(B>A\;[95\%\;CI:-0.136--0.043])$, see Fig. 6a. However, although *Model A* exhibits a higher PR-AUC than *Model B*, the difference was not significant for *DEEP-HPA*: $\bar{x}=0.012,\;p<0.094;(\mathrm{Unclear}\;\mathrm{direction}\;[95\%\;CI:-0.001-0.031])$, see Fig. 6b.

**Figure 7:**
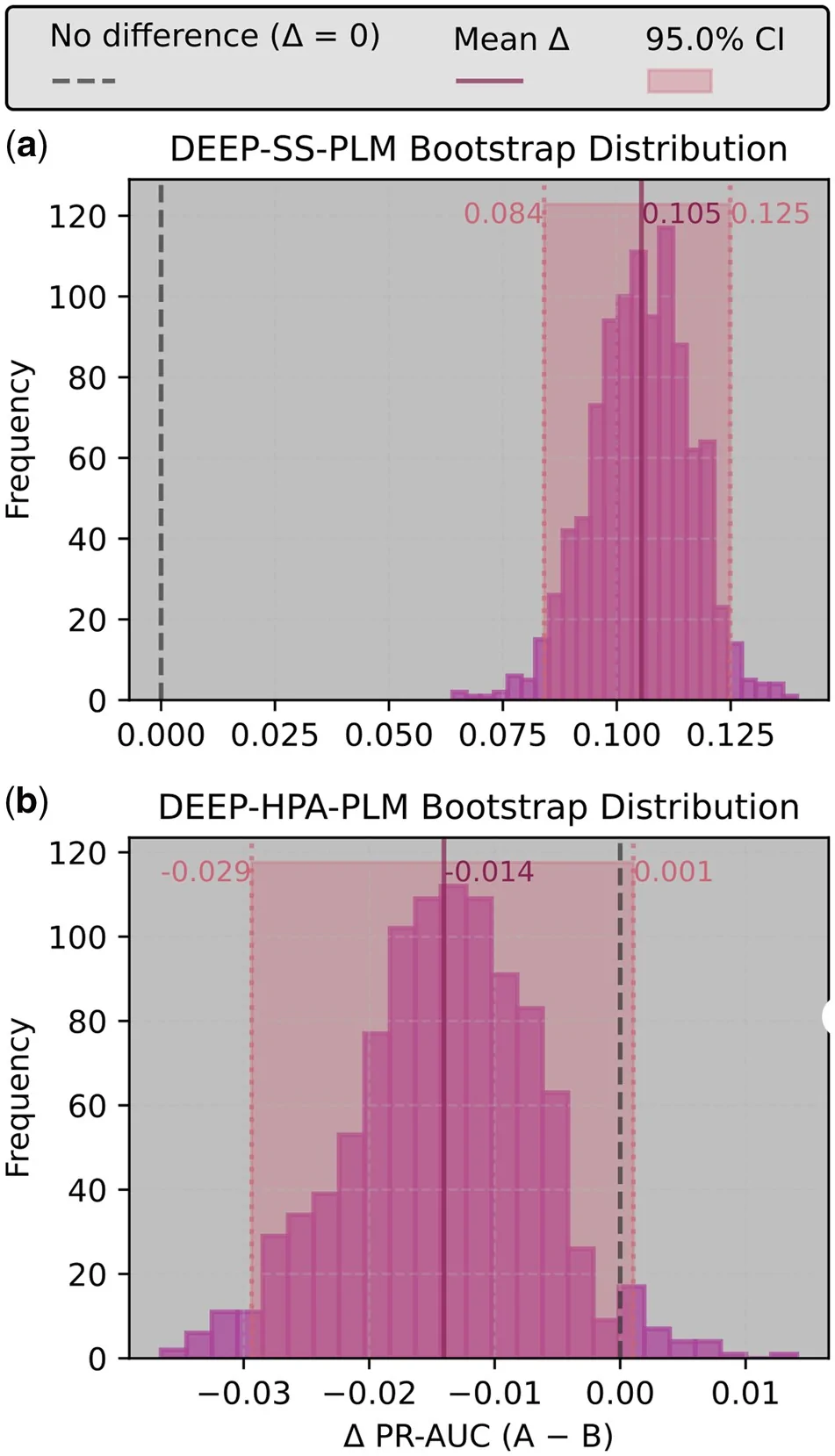
Histogram of the distribution of 1000 bootstrap PR-AUC metric differences between PLM-based models *A* and *B* as trained on *SCL2205*${\;}_{s}$ and *DEEP-TV*${\;}_{s}$, respectively. The top panel **(a)** was tested on *DEEP-SS* (A>B), whereas the bottom panel **(b)** was tested on (*DEEP-HPA*) (no significant difference).

*PLM-based architecture* Similar to the above architecture, we implemented testing on the PLM-based models. Interestingly here, we observed the reverse trend for *DEEP-SS*: $\chi^{2}(df=1)=67.0,\;p<0.001;\;(A>B\;[b=90,c=8])$ with an Effect Size Z=8.3, while the difference was not significant for *DEEP-HPA*: $\chi^{2}(df=1)=3.5,\;p<0.060;\;(\mathrm{Unclear}\;\mathrm{direction}\;[b=109,c=82])$ with an Effect Size Z=2.0. In contrast, for the stratified bootstrap on the PR-AUC metric difference test (Δ=A−B) for *DEEP-SS*: $\bar{x}=0.105,\;p<0.001;\;(A>B\;[\;95\%\;CI:0.084-0.125])$, see Fig. 7a. Nonetheless, despite *Model B* depicting a higher PR-AUC than *Model A*, the difference was not significant, just as before; *DEEP-HPA*: $\bar{x}=-0.014,\;p<0.07;\;(\mathrm{Unclear}\;\mathrm{direction}\;[95\%\;CI:-0.029-0.001])$, see Fig. 7b.

*Model heterogeneity* Evidence of heterogeneous model behaviour, assessed using *SCL2205*, was observed in independent test sets. For the *DEEP-SS* subset, Cochran’s *Q* was 53.06 (heterogeneity *P*-value, $p_{\mathrm{het}}=3.24\times 10^{-13}$). For the (*DEEP-HPA*) subset, Cochran’s *Q* was 268.5 ($p_{\mathrm{het}}<0.001$). In both cases, the null hypothesis of homogeneity was rejected, indicating statistically significant heterogeneity in the distribution of PR-AUC deltas between the models.

Notably, the performance of the training data seemed to be influenced by the model architecture. PLM-based models had lower uncertainty compared to CNN-based models; Figs 7 and 6.

### 4.4 The final SCL2205 dataset

We propose *SCL2205*, a leakage-minimized dataset for SCL modelling that incorporates expert manual curation. This approach ensures high-quality training input, thereby favouring efficient model development. The specific compositions for the two tracks of the *SCL2205* dataset (n=19074) are provided in File 1—*Results*, available as supplementary data at *Bioinformatics* online. Furthermore, we provide a convenient open-source interface (p-scldata) to facilitate easy access to the dataset. This is publicly available in Python Package Index (PyPI), ensuring that *SCL2205* is easily integrated into existing AI/ML and bioinformatics workflows.

## 5 Discussion

The rapid advancement of AI in genomics has created an urgent need for robust and standardized datasets. *SCL2205* addresses this gap by providing a carefully curated resource for sequence-based spatial modelling. By implementing a stringent homology-reduction procedure, we minimize overlap between training, evaluation, and test sets, thereby reducing the risk of information leakage and inflated performance estimates that might plague current SCL predictors (Urban *et al.* 2020, Whalen *et al.* 2021). This work introduces a new and transparent benchmark foundation for the development and evaluation of modern AI-based spatial protein predictors, including PLM-based approaches.

Because high sequence similarity is often associated with functional similarity (Nair and Rost 2002), homology can be exploited for function transfer when a suitable reference protein is available. However, such a transfer is inherently imperfect, and many proteins lack sufficiently similar annotated references (Higdon *et al.* 2010). In real-world scenarios, biological sequence databases contain substantial redundancy, while experimentally characterized proteins remain relatively scarce. Although homology reduction may reduce opportunities for function transfer between highly similar sequences, these cases arguably represent the simpler prediction tasks. The more challenging and biologically relevant task is to annotate proteins with limited similarity to characterized references. By reducing dependence on close homologues, homology reduction encourages the development of models that generalize beyond highly redundant sequence clusters. Ultimately, effective predictors should balance the benefits of homology-aided transfer with the need to accurately predict more distant and poorly annotated proteins, which comprise the majority in sequence databases.

Inherently, DL modelling requires large-scale data for training. Although true, studies have shown that smaller, carefully curated datasets can improve model performance (Marion *et al.* 2023). Although the pre-training data sources in NLP are comparatively less controlled than UniProtKB, the latter still contains poor-quality data that requires quality control and assurance preprocessing.

Problems in the life sciences are often critical and require more stringent performance controls. Therefore, we applied a variety of filters; importantly, careful manual curation, to ensure high-quality pre-training SCL data. For instance, unlike in (Armenteros *et al.* 2017) and (Thumuluri *et al.* 2022), where the UniProtKB annotation quality scores are ignored, probably because they reflect the overall sequence annotation (not SCL only), we also filter based on the annotation score. A distribution for the scores is summarized in Fig. 1, available as supplementary data at *Bioinformatics* online. Applying an annotation-score threshold of ≥3 excluded 1377 (4.3%) sequences, comprising 90 (0.3%) and 1287 (4.0%) sequences with annotation scores of one and two, respectively; while a threshold of ≥4 would exclude an additional 3625 (11.2%) sequences. By considering annotation quality scores, we emphasize protein functional interrelationships as inseparable biological processes and expect the chosen threshold to support better dataset reliability. Similarly, it may also be argued that protein functional annotation is progressive and that certain aspects of a protein may be better understood than others at any given time. However, highly beneficial insights can be obtained from a broadly understood basis, as reflected in the overall annotation quality score, thereby promoting discovery.

Another key technical distinction of the *SCL2205* dataset is our decision to retain a sequence cut-off of up to 5000 amino acids, avoiding the common practice of aggressive truncation. Although most SoTA predictors truncate sequences to 1000 residues to reduce computational overhead, such choices risk discarding critical biological information. Our approach is premised on signal positional agnosticism; we assume that SCL signals—such as C-terminal *ER*-retention motifs [e.g. Lys-Asp-Glu-Leu (KDEL)] or internal nuclear localization signals—can occur anywhere within the protein primary structure.

By preserving nearly the entire sequence length distribution of the original UniProtKB records, *SCL2205* ensures that bidirectional architectures, including bidirectional long-short-term memory (bi-LSTMs) and the latest PLMs, can leverage terminal information from both the *N*- and *C*-termini. Truncation at the 1000-residue mark would effectively ‘blind’ the backward pass of a bi-LSTMs or the global attention mechanism of a *Transformer* to the C-terminal context of larger proteins. Consequently, *SCL2205* provides a more biologically consistent representation, particularly for the notable fraction of the eukaryotic proteome that exceeds standard truncation limits, thus mitigating the risk of false negatives for locations defined by non-amino-terminal signals.

SCL datasets are inductively imbalanced, which contributes to the omission of low-representation classes from model development, as evidenced by some DL predictors (Armenteros *et al.* 2019, Kaleel *et al.* 2021). With low target diversity, generalization is bound to suffer. Therefore, although exhibiting the potential for a precision-generalization trade-off, we alleviated the problem by manual label mapping (Fig. 2).

Notably, from Table 1, the number of examples for certain locations—such as the *Membrane*—drastically increased. A category like *Plastid* would be excluded from modelling by, say, a mapping logic just considering the term ‘plastid’, which is broadly defined in uniprotkb’s SCL ontology.

To highlight the mapping process, we use a bar chart (Fig. 4) to illustrate the exact mapping for *Plastid—*the most enriched location—as a representative case. When considered alongside the individual definitions from *Gene Ontology and GO Annotations*, we can justify the observed logic. For example, a *Plastid* is defined as: ‘*Any member of a family of organelles found in the Cytoplasm of plants and some protists, which are membrane-bounded and contain DNA.’*

However, nuanced cases sometimes require a ‘voting’ strategy, which risks being subjective, especially if not anchored on established domain knowledge. For instance, in the case of the multi-label *Chloroplast stroma; Chloroplast thylakoid membrane; Plastid*, the majority of labels favoured a generalized mapping to *Plastid* instead of *Membrane*. In instances where a ‘draw’ occurred between two locations of interest, such as *Chloroplast thylakoid membrane; Plastid*, we considered that mapping to *Membrane* was consistent with a more definitive biological classification. However, it is also justifiable that mapping to *Plastid* supports a broader generalization. Such rare edge cases inevitably involve trade-offs and may represent an inherent limitation of mapping-based data augmentation. Nonetheless, benchmarks such as *SCL2205* provide an essential foundation, enabling consistency and standardization across diverse studies, which fosters comparability as mapping conventions mature.

Consequently, manual intervention, though labour-intensive, not only increased the number of training examples but also improved class diversity. This provides a dataset that closely reflects the diversity expected in real-world applications and may support enhanced generalization, including to the *dark proteome* or OOD scenarios. As demonstrated by our results, when the dataset is coupled with a complementary model architecture (see Figs 6 and 7 in Section 4), enhanced model performance is most apparent. Furthermore, although location imbalance persists, it has been substantially reduced (see Table 1). Therefore, *SCL2205* provides a solid foundation for further augmentation mechanisms, particularly during model development. We are currently exploring these avenues and encourage the community to build on *SCL2205* in tackling similar directions.

Our label-mapping results suggest a compelling enhancement to model ranking quality, especially for the PLM-based model, while underscoring the complexity of the overfitting-versus-generalization trade-off in spatial modelling. For evaluation rigour, two statistical tests were used: McNemar’s test and stratified bootstrapping CI on PR-AUC differences. While the PR-AUC comparison determines which model better ranks and retrieves positives across various thresholds (quality), McNemar’s test evaluates which model performs better at a fixed decision threshold (the decision rule). Compared to AUROC, which tends to bias performance towards majority classes, we opted to use PR-AUC as it is imbalance-aware (Saito and Rehmsmeier 2015).

In the case of *DEEP-SS*, the significant performance boost (macro PR-AUC: 9.0% points) strongly suggests that extensive label mapping helped the model identify generalizable localization patterns. However, the results for (*DEEP-HPA*) tell a conflicting story across the tests. Based on McNemar’s test, the original labels (*Model B*) outperformed mapped labels (*Model A*), whilst the opposite was observed with the bootstrapping test, indicating that improved ranking performance did not translate into superior hard classification performance at a fixed operating point. With reference to McNemar’s test, the discrepancy reflects two things: first, that the datasets contain different localization signals; and secondly, that in a *head-to-head* consideration of disagreements between the two models, *Model B* produced more correct predictions. But this is not entirely unexpected for *DEEP-HPA*, because the taxonomic fidelity associated with the human protein atlas data may limit the extent of extractable spatial signal, or the extractable signal may align better with certain signal learners than others. These phenomena were particularly true for local signal extractors such as CNNs, as demonstrated in the results (see Section 4.3) for the experiment in Section 3.3.3, isolating model effects.

We also evaluated a current SoTA augmentation approach, homology augmentation (see Section 2.1 for definition), to assess its impact on data partitioning. Previous studies have demonstrated the benefits of incorporating evolutionary information into both structure prediction (Baek *et al.* 2021, Jumper *et al.* 2021) and SCL prediction (Nair and Rost 2002, Adelfio *et al.* 2013). These benefits may arise from substantial functional conservation, as shown by Nair and Rost (2002), who observed that SCL could be transferred with up to 90% accuracy for proteins with as little as 50% sequence identity.

Interestingly, their findings also indicated that SCL transfer remains feasible even at lower identity thresholds, consistent with observations in structure prediction (Mooney and Pollastri 2009), particularly for major SLs, which are overrepresented in databases. Despite these advantages, certain issues are either perpetuated or newly introduced. Although class imbalance is inherent, data leakage remains a critical concern, especially in SoTA scenarios that claim ‘stringent *training–testing* data partitioning’. We conjectured residual leakage due to homology augmentation, especially for classification, where sequence labels are inherent, despite using unlabelled sequences for augmentation.

While testing this hypothesis is primarily complicated by the intensive resource requirements for homology searches, we applied 10 bootstrap samples to assess its possible implications (homology search took over 150 wall-clock-days for approx. 800 sequences). Homology augmentation is standard practice in sequence-based DL SCL predictors. It is based on conserving evolutionary function and has been shown to improve prediction performance relative to *sequence-feature-based-only* approaches (Nair and Rost 2002, Gillani and Pollastri 2025). Although it can amplify signals using profiles of homologous proteins, common implementations have been shown to bias model evaluation (Urban *et al.* 2020). The bias results from the overlap between the training and testing datasets (data leakage).

Our findings demonstrated that data leakage of at least 5.2% occurred when the same overlap-reduction (partitioning) approach applied prior to homology augmentation was used, even with only 10% of the examples in the training set used for homology augmentation. In contrast, previous efforts to examine biases in comparing profile- and non-profile-based SCL predictors, (Urban *et al.* 2020), used an *ad hoc* sequence similarity proxy that differed from those used for data partitioning in the models considered, potentially obscuring the true extent of overlap.

Theoretically, our findings suggest that achieving 100% data leakage would require fewer examples than the full training set; however, this would depend on factors such as the degree of homology in the original dataset and the extent of overlap between the training/testing sets, and the homology augmentation database. Regardless, we hypothesize that, relative to common practices in homology augmentation, such as MSA and position-specific scoring matrices, the extent of data leakage would be even greater than that observed with our 10% subset of native sequence data. Whereas our sequence similarity comparisons are conducted on a native-to-native, sequence-to-sequence basis, MSA- and PSSM-based approaches typically compare an averaged (consensus) sequence against a native sequence. Because a consensus sequence is intrinsically more likely to produce a broader range of hits, this implies greater overlap and, consequently, increased leakage, potentially even across different subcellular locations.

Therefore, at a minimum, predictors applying homology reduction followed by homology augmentation must assess and report any post-homology-augmentation *training–testing* dataset overlap. We acknowledge that rigorously evaluating this phenomenon may be challenging, as the sequence representation following homology augmentation often differs from that used during initial homology reduction unless equivalent consensus sequences are used.

In light of the above, emerging alternative augmentation strategies, such as leveraging PLMs (Heinzinger *et al.* 2019, Rives *et al.* 2021, Elnaggar *et al.* 2022, Thumuluri *et al.* 2022), may lead to better results with further research. Interestingly, we observed that *SCL2205* better complements such new frontiers of PLMs by exhibiting improved generalization over *DEEP-TV* for the *in-distribution* set (*DEEP-SS*; Fig. 7). To delineate true effect from the noise, we performed a heterogeneity sanity check using Cochran’s *Q* test. The significant heterogeneity observed for the models (see Section 4.3) suggests that the CNN and PLM architectures are learning fundamentally different signals from the same data. This seems consistent with what may be considered as unique ‘specialities’ for these models; CNNs are local learners (*k*-window kernels), while PLMs are both local and global learners (attention along the entire sequence).

Interestingly, we observed contrasting results between *SCL2205* and *DEEP-TV* on both independent test sets when using the CNN-based model (Fig. 6). These differences may be attributed to several factors beyond architectural design. Firstly, with respect to the evaluation sets, the *DEEP-HPA* set exhibits a distinct taxonomic bias, comprising exclusively human proteins, whereas *DEEP-SS* comprises proteins from multiple species. Consequently, both *SCL2205* and *DEEP-TV*, which are themselves multi-species datasets, may reduce the relative contribution of human-specific signals during training. However, the manual label-mapping divergence between the two datasets—the former more extensive than the latter in label aggregation—implies a better generalization for *SCL2205* than *DEEP-TV*. Notably, this effect appears to depend on the underlying model architecture (see Section 4.3). Secondly, on the architectures, the training paradigm for PLMs appears to align more naturally with our label-mapping approach. PLM training uses large-scale, general, unlabelled corpora, enabling the learning of broad biological representations that are expected to support generalization. In contrast, CNNs operating on non- PLM embeddings probably suffer from limited signal diversity, potentially constraining their ability to benefit from the increased label diversity introduced through manual mapping.

From a training-data perspective, we expect datasets with limited signal diversity to favour models that rely primarily on local sequence patterns (e.g. CNN), particularly when evaluated on in-distribution data. Conversely, datasets with greater signal diversity are expected to benefit more from architectures that capture local and global sequence dependencies (e.g. PLMs), thereby improving generalization to OOD data. More importantly, our findings suggest that model architecture can influence how effectively information contained within the training data is utilized. In particular, the strong performance of a limited-signal dataset when paired with a local–global signal learner highlights the robustness of PLMs. Coherent with this interpretation, our model-effect isolation experiments supported these observations. The results also corroborate previous studies (Stärk *et al.* 2021, Thumuluri *et al.* 2022).

Finally, differences in model calibration can contribute to overconfidence, particularly in PLMs (Guo *et al.* 2017, Desai and Durrett 2020, Chen et al. 2023). However, both models were tested in their *vanilla* forms, without calibration or optimization, and reliability diagrams depicted very similar profiles across the model variants.

These divergences highlight a critical trade-off in spatial modelling: the delicate balance between breadth (e.g. through mapping and/or taxonomic diversity) and depth (the precision of native labels and/or taxonomic specificity).

Overall, *SCL2205* aligns more effectively than *DEEP-TV* with the increasing adoption of pre-trained PLMs in the AI-genomics frontiers. This distinction is critical for the PLM frontier: if a researcher requires a definitive ‘yes/no’ prediction, the native labelling of *Model B* is preferable. For large-scale genomic annotation prioritizing ranking predictions, *SCL2205* (*Model A*) is the data of choice. Similarly, *SCL2205* is better for *hard-to-predict* examples, while retaining satisfactory performance on *easy-to-predict* examples. Given the relative ease with which highly homologous sequences can be inferred through annotation transfer (Nair and Rost 2002), the main goal of SCL predictors, which homology reduction helps with, is to predict sparsely represented examples. *SCL2205* particularly suits this goal.

Ultimately, we have proposed a rigorously developed dataset that improves the trustworthiness and robustness of the AI SCL modelling. Additionally, it facilitates efficient and environmentally sustainable development by mitigating ‘noisy’ overhead data, whilst enriching generalization through diversity. By providing *SCL2205* as an installable Python package (p-scldata), we adhere to open-science principles, ensure seamless integration with modern ML frameworks, and provide a compelling benchmark for future genomic discovery. In future work, we aim to develop an automated end-to-end pipeline that continuously updates *SCL2205* with newer UniProtKB releases, thereby ensuring its long-term relevance and utility.

## 6 Conclusion

The computational annotation of proteins with SCL remains a critical component of functional genomics. As the field enters new frontiers, such as PLMs, new challenges emerge; most notably in the quality and diversity of training input data. Whereas data are the primary force behind the AI revolution, acquiring high-quality datasets for training biological models remains a significant hurdle. Nevertheless, techniques such as data augmentation and human-aided curation have begun to alleviate these issues.

In this study, we present *SCL2205—*a new high-quality dataset that highlights the limitations of current state-of-the-art approaches to data development. Through rigorous independent evaluation, we demonstrate that *SCL2205* is an improvement on existing alternatives, particularly for applications involving PLM-based modelling. We acknowledge that certain persistent challenges, such as class imbalance, must still be addressed through further research beyond data preparation. Nevertheless, *SCL2205* serves as a trustworthy and reliable benchmark for AI application in SCL prediction, laying the foundation for future discoveries in the genomic landscape. Ultimately, by providing a more accurate spatial map of localization within the cell, we move closer to a future where we can rapidly identify the molecular drivers of rare diseases and accelerate the development of life-changing targeted therapies.

## Supplementary Material

btag666_Supplementary_Data

## Data Availability

Data are available through the following open-source avenues: **DRYAD** (Data): an archive (https://doi.org/10.5061/dryad.2ngf1vj1t) under the Creative Commons Zero (CC0 1.0) licence. **PyPI** (Software): an installable Python data package (p-scldata [Version 2026.2.0]) under the MIT licence. **Repository** (Data & Code): https://github.com/ousodaniel/scldata/blob/main (Checkpoint f4e9ac7) and **Zenodo** (https://doi.org/10.5281/zenodo.21796423).

## References

[btag666-B1] Adelfio A , Volpato V, Pollastri G. Sclpredt: ab initio and homology-based prediction of subcellular localization by n-to-1 neural networks. Springerplus 2013;2:502–11. 10.1186/2193-1801-2-502 PMC3795874 24133649

[btag666-B2] Armenteros JJA, Sønderby CK, Sønderby SK et al Deeploc: prediction of protein subcellular localization using deep learning. Bioinformatics 2017;33:4049. 10.1093/BIOINFORMATICS/BTX548 29028934

[btag666-B3] Armenteros JJA, Salvatore M, Emanuelsson O et al Detecting sequence signals in targeting peptides using deep learning. Life Sci Alliance 2019;2. 10.26508/LSA.20190042 PMC6769257 31570514

[btag666-B4] Baek M , DiMaio F, Anishchenko I et al Accurate prediction of protein structures and interactions using a three-track neural network. Science 2021;373:871–6, 8. 10.1126/science.abj8754 PMC7612213 34282049

[btag666-B5] Blum T , Briesemeister S, Kohlbacher O. Multiloc2: integrating phylogeny and gene ontology terms improves subcellular protein localization prediction. BMC Bioinformatics 2009;10:274–9. 10.1186/1471-2105-10-274 PMC2745392 19723330

[btag666-B6] Chen Y, Geoff P, Yu S et al A close look into the calibration of pre-trained language models. In: Rogers A, Boyd-Graber J, Okazaki N (eds.), *Proceedings of the 61st Annual Meeting of the Association for Computational Linguistics* (Volume 1: Long Papers), Toronto, Canada: Association for Computational Linguistics, 2023, 1343–67. 10.18653/v1/2023.acl-long.75

[btag666-B7] Desai S , Durrett G. Calibration of pre-trained transformers. In: Webber B et al (eds.), Proceedings of the 2020 Conference on Empirical Methods in Natural Language Processing (EMNLP). Punta Cana, Dominican Republic: Association for Computational Linguistics, 2020, 295–302. 10.18653/v1/2020.emnlp-main.21

[btag666-B8] Elnaggar A , Heinzinger M, Dallago C et al Prottrans: toward understanding the language of life through self-supervised learning. IEEE Trans Pattern Anal Mach Intell 2022;44:7112–27. 10.1109/TPAMI.2021.3095381 34232869

[btag666-B9] Fan K , Wang W. What is the minimum number of letters required to fold a protein? J Mol Biol 2003;328:921–6. 10.1016/S0022-2836(03)00324-3 12729764

[btag666-B10] Feng SY, Gangal V, Wei J et al A survey of data augmentation approaches for nlp. In: Findings of the Association for Computational Linguistics. Virtual: ACL-IJCNLP 2021, 2021, 968–88. 10.18653/V1/2021.FINDINGS-ACL.84

[btag666-B11] Fu L , Niu B, Zhu Z et al Sequence analysis cd-hit: accelerated for clustering the next-generation sequencing data. Bioinformatics 2012;28:3150–2. 10.1093/bioinformatics/bts565 PMC3516142 23060610

[btag666-B12] Gillani M , Pollastri G. Impact of alignments on the accuracy of protein subcellular localization predictions. Proteins Struct Funct Bioinf 2024;93:745–59. 10.1002/PROT.26767 PMC11809130 39575640

[btag666-B13] Guo C, Geoff P, Yu S et al On calibration of modern neural networks. In: Proceedings of the 34th International Conference on Machine Learning, Volume 70 of ICML’17. Sydney, NSW, Australia: Journal of Machine Learning Research, 2017, 1321–30. 10.5555/3305381.3305518

[btag666-B14] Heinzinger M , Elnaggar A, Wang Y et al Modeling aspects of the language of life through transfer-learning protein sequences. BMC Bioinformatics 2019;20:723–14712105. 10.1186/S12859-019-3220-8 PMC6918593 31847804

[btag666-B15] Higdon R , Louie B, Kolker E. Modeling sequence and function similarity between proteins for protein functional annotation. Proc Int Symp High Perform Distrib Comput 2010;2010:499–502. 10.1145/1851476.1851548 PMC4120521 25101328

[btag666-B16] Höglund A , Dönnes P, Blum T et al Multiloc: prediction of protein subcellular localization using n-terminal targeting sequences, sequence motifs and amino acid composition. Bioinformatics 2006;22:1158–65. 10.1093/bioinformatics/btl002 16428265

[btag666-B17] Jumper J , Evans R, Pritzel A et al Highly accurate protein structure prediction with alphafold. Nature 2021;596:583–9. 10.1038/s41586-021-03819-2 PMC8371605 34265844

[btag666-B18] Kaleel M , Ellinger L, Lalor C et al Sclpred-mem: subcellular localization prediction of membrane proteins by deep n-to-1 convolutional neural networks. Proteins Struct Funct Bioinf 2021;89:1233–9. 10.1002/PROT.26144 33983651

[btag666-B19] Kaleel M , Zheng Y, Chen J et al Sclpred-ems: subcellular localization prediction of endomembrane system and secretory pathway proteins by deep n-to-1 convolutional neural networks. Bioinformatics 2020;36:3343–9. 10.1093/bioinformatics/btaa156 32142105

[btag666-B20] Kudo T. Subword regularization: Improving neural network translation models with multiple subword candidates. In: Gurevych I, Miyao Y (eds.), Proceedings of the 56th Annual Meeting of the Association for Computational Linguistics (Volume 1: Long Papers), Melbourne, Australia: Association for Computational Linguistics, 2018. 66–75. 10.18653/v1/P18-1007

[btag666-B21] LeCun Y , Cortes C. The MNIST database of handwritten digits. 2010.

[btag666-B22] Maas AL, Daly RE, Pham PT et al Learning word vectors for sentiment analysis. In: Lin D, Matsumoto Y, Mihalcea R (eds.), *Proceedings of the 49th Annual Meeting of the Association for Computational Linguistics: Human Language Technologies*, Volume 1 of *HLT ’11*. Portland, Oregon, USA: Association for Computational Linguistics, 2011, 142–50. 10.5555/2002472.2002491

[btag666-B23] Marion M, Üstün A, Pozzobon L et al When less is more: investigating data pruning for pretraining LLMS at scale, arXiv, arXiv:2309.04564, 2023, preprint: not peer reviewed.

[btag666-B24] Mercier PL, Bolleman J, Castro ED et al Swissbiopics—an interactive library of cell images for the visualization of subcellular location data. Database J Biol Databases Curation 2022:2022:1–5. 10.1093/DATABASE/BAAC026 PMC9216577 35411389

[btag666-B25] Mooney C , Pollastri G. Beyond the twilight zone: automated prediction of structural properties of proteins by recursive neural networks and remote homology information. Proteins Struct Funct Bioinf 2009;77:181–90. 10.1002/PROT.22429 19422056

[btag666-B26] Nair R , Rost B. Sequence conserved for subcellular localization. Protein Sci 2002;11:2836–47. 10.1110/PS.0207402 PMC2373743 12441382

[btag666-B27] Rives A , Meier J, Sercu T et al Biological structure and function emerge from scaling unsupervised learning to 250 million protein sequences. Proc Natl Acad Sci USA 2021;118:e2016239118. 10.1073/pnas.2016239118 PMC8053943 33876751

[btag666-B28] Rost B. Twilight zone of protein sequence alignments. Protein Eng 1999;12:85–94. 10.1093/PROTEIN/12.2.85 10195279

[btag666-B29] Saito T , Rehmsmeier M. The precision-recall plot is more informative than the roc plot when evaluating binary classifiers on imbalanced datasets. PLoS One 2015;10:e0118432. 10.1371/JOURNAL.PONE.0118432 PMC4349800 25738806

[btag666-B30] Shorten C , Khoshgoftaar TM. A survey on image data augmentation for deep learning. J Big Data 2019;6:1–48. 10.1186/s40537-019-0197-0 PMC8287113 34306963

[btag666-B31] Shorten C , Khoshgoftaar TM, Furht B. Text data augmentation for deep learning. J Big Data 2021;8:101. 10.1186/s40537-021-00492-0 PMC8287113 34306963

[btag666-B32] Stärk H , Dallago C, Heinzinger M et al Light attention predicts protein location from the language of life. Bioinform Adv 2021;1:vbab035. 10.1093/bioadv/vbab035 PMC9710637 36700108

[btag666-B33] Thul PJ , Åkesson L, Wiking M et al A subcellular map of the human proteome. Science (1979) 2017;356:10959203. 10.1126/science.aal3321 28495876

[btag666-B34] Thumuluri V , Almagro Armenteros JJ, Johansen AR et al Deeploc 2.0: multi-label subcellular localization prediction using protein language models. Nucleic Acids Res 2022;50:W228–34. 10.1093/nar/gkac278 PMC9252801 35489069

[btag666-B35] UniProtConsortium, Bateman A, Martin MJ et al Uniprot: the universal protein knowledgebase in 2023. Nucleic Acids Res 2023;51:D523–31. 10.1093/NAR/GKAC1052 PMC9825514 36408920

[btag666-B36] Urban G , Torrisi M, Magnan CN et al Protein profiles: biases and protocols. Comput Struct Biotechnol J 2020;18:2281–9. 10.1016/J.CSBJ.2020.08.015 PMC7486441 32994887

[btag666-B37] Walsh I , Fishman D, Garcia-Gasulla D et al; ELIXIR Machine Learning Focus Group. Dome: recommendations for supervised machine learning validation in biology. Nat Methods 2021;18:1122–7. 10.1038/s41592-021-01205-4 34316068

[btag666-B38] Whalen S , Schreiber J, Noble WS et al Navigating the pitfalls of applying machine learning in genomics. Nat Rev Genet 2021;23:169–81. 10.1038/s41576-021-00434-9 34837041

[btag666-B39] Wolf T, Debut L, Sanh V et al Transformers: state-of-the-art natural language processing. In: Proceedings of the 2020 Conference on Empirical Methods in Natural Language Processing: System Demonstrations. Virtual: Association for Computational Linguistics, 2020; 38–45. 10.48550

