# Supplementary material for: Developing SCL2205: a protein sequence-based spatial modelling dataset for the protein language model frontier
Source: Bioinformatics. 2026 Sep 18;42(9):btag666. doi: 10.1093/bioinformatics/btag666 (PMC13616090; doi:10.1093/bioinformatics/btag666)
Supplement: Supplementary file 1 [file btag666_supplementary_data.zip › supplementary-file-s1.pdf]

# Supplementary Methodology

## Annotation score distribution

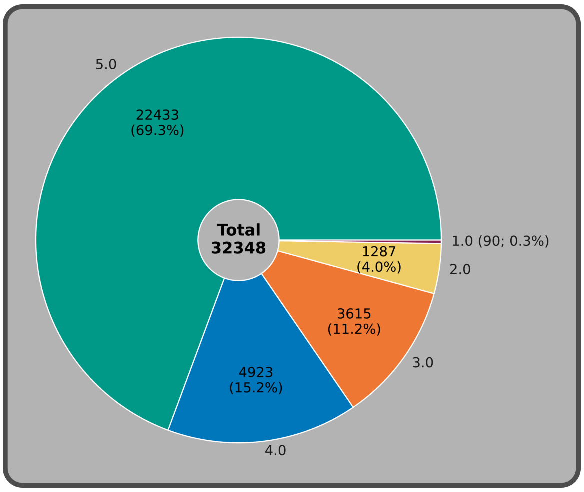


**Fig. 1. Summary of annotation scores**. The summary followed the application of four of the five filtering criteria: presence of subcellular localisation (SCL) annotation, taxonomy, experimental evidence, and sequence length ($n = 32\,348$ sequences).
**Alt text**: Doughnut chart showing the distribution of sequences across five annotation-score categories after quality filtering. Most sequences are associated with annotation scores of 3 or higher, while a smaller proportion have scores of 1 or 2 and were excluded from subsequent analyses.

## Manual label mapping dictionary

A **Supplementary File S2; Table 1** contains the manual mapping and harmonisation of SCL labels.

## Taxonomic composition

**Table 1. Distribution of data across kingdoms and preparation stages**: *PrpS0* (22 152 proteins) and *PrpS1* (19 074 proteins; the ***SCL2205*** dataset), captured in **Fig. 2** of the main text. The columns contain the taxonomic classification with data preparation stage sub-columns. The first and second rows are counts and percentages, respectively.

| **Kingdom** | **Metazoa** | | **Viridiplantae** | | **Fungi** | | **Other *** | |
|---|---|---|---|---|---|---|---|---|
| **Prep stage** | *PrpS0* | *PrpS1* | *PrpS0* | *PrpS1* | *PrpS0* | *PrpS1* | *PrpS0* | *PrpS1* |
| **Counts** | 13 390 | 10 786 | 4250 | 3857 | 4198 | 4137 | 314 | 294 |
| **Percentage** | 60.45% | 56.55% | 19.19% | 20.22% | 18.95% | 21.69% | 1.42% | 1.54% |

* No taxonomy assignment at the *Kingdom* level.

## Sequence alignments

We used PSI-BLAST for pairwise *all-against-all* alignments and `makeblastdb` to generate search databases, with the parameters outlined below. Unaccounted parameters were used with their default values.

Database Generation

```
makeblastdb  -parse_seqids \
    -dbtype ‘‘prot’’ \
    -in <File_fasta> \ # variable  depending on input
    -out <str, in> \ # variable depending on input
    -title <String> # variable depending on input
```

Pairwise Alignment

```
psiblast -query <query_file> \ # variable depending on input
    -db <str> \ # variable depending on input
    -out <out_file> \ # variable depending on input
    -evalue 0.005 \
    -word_size <2=<int<=5> \ # variable depending on similarity threshold
    -matrix BLOSUM62 \
    -outfmt ''10 delim=@ qseqid qlen sseqid slen qstart qend sstart send qseq \
            sseq evalue length mismatch gaps pident nident'' \
    -max_target_seqs 1500 \
    -num_iterations 1 \ # default
    -num_threads <Integer, >=1> # variable depending on the number of cores in the hardware
```

Word size depended on the similarity threshold for homology reduction, similar to the *CD-HIT* tool (Fu et al. 2012), as follows.

```
if isinstance(p_ident, int):
    if 0 <= p_ident < 50:
        word_size = 2
    elif 50 <= p_ident < 60:
        word_size = 3
    elif 60 <= p_ident < 70:
        word_size = 4
    elif 70 <= p_ident <= 100:
        word_size = 5
```

Throughout, we set the number of iterations to one, effectively using position-specific iterative BLAST (PSI-BLAST) in its BLASTP-equivalent mode. Using more than one iteration would identify distant homologues, which we intentionally avoided. This choice was motivated by two main considerations:

1. The homology paradox – while some relatedness is necessary for learning, excessive similarity can be detrimental. Iterative PSSM can overly reduce homology.
2. Data leakage – including distant homologs during homology augmentation could increase residual overlap boundary between datasets that were initially *overlap-reduced.*

## Similarity algorithm

We calculated three identity percentages from the resulting alignments using a common numerator – the number of identical amino acids in the alignment – but distinct denominators as follows:

1. Length of the shortest sequence in the pairwise alignment
2. Length of the longest sequence in the pairwise alignment
3. Length of the *no-gap* alignment

Subsequently, for the final percentage similarity score $S$, the weighted geometric mean identity was computed, using the `scipy.stats` module, while penalising the longest sequence by applying its complement weight $(1 - w_l^{seq})$; where $w_l^{seq}$ is the actual weight of the longest sequence. We applied this penalty because percent identity is bounded by the longest sequence, that is, it dictates the maximal possible identity within the alignment *system*. This approach reduces the number of tunable hyperparameters compared to tools such as *CD-HIT*, and eliminates the need for length-based sequence ordering prior to homology reduction.

## Dataset compositions

*Label mapping training-testing preprocessing*

**Table 2.** Effect of independent test set processing before testing, with respect to with and without label mapping evaluations. **Original**: Full independent test set size. **No overlap**: Samples remaining after overlap reduction with the training datasets. **Stats valid**: Samples remaining after ensuring validity for McNemar's and bootstrapping confidence interval (CI) paired statistical comparison.

| **Subcellular location** | **DEEP-SS** | | | **DEEP-HPA** | | |
|---|---|---|---|---|---|---|
| | **Original** | **No overlap** | **Stats valid** | **Original** | **No overlap** | **Stats valid** |
| **Label Mapping** | | | | | | |
| Secreted† | 522 | 184 | 184 | NA | NA | NA |
| Membrane | 170 | 108 | 108 | 161 | 149 | 149 |
| Mitochondrion | 111 | 74 | 74 | 175 | 154 | 154 |
| Peroxisome | 77 | 30 | 30 | 7 | 7 | 7 |
| Nucleus | 63 | 43 | 43 | 664 | 622 | 622 |
| Cytoplasm | 10 | 6 | 6 | 263 | 249 | 249 |
| ER | 5 | 4 | 4 | 31 | 29 | 29 |
| **Totals** | 1301 | 1210 | 1210 | 1301 | 1210 | 1210 |
| **No Label Mapping** | | | | | | |
| Secreted† | 522 | 189 | 184 | NA | NA | NA |
| Membrane | 170 | 165 | 108 | 161 | 159 | 149 |
| Mitochondrion | 111 | 82 | 74 | 175 | 161 | 154 |
| Peroxisome | 77 | 31 | 30 | 7 | 7 | 7 |
| Nucleus | 63 | 47 | 43 | 664 | 633 | 622 |
| Cytoplasm | 10 | 7 | 6 | 263 | 255 | 249 |
| ER | 5 | 4 | 4 | 31 | 31 | 29 |
| **Totals** | 1301 | 1246 | 1210 | 1301 | 1246 | 1210 |

† Secreted class absent in **DEEP-HPA**.

*Dataset comparisons training-testing preprocessing*

*Software and tools information*

`Software specifications`

1. Python: 3.11
2. JupyterLab: 4.1.5
3. BLAST: 2.13.0, build Feb 2 2022 15:38:31
4. RefSeq: BLASTDB Version 5, Oct 26 2024 05:37
5. Pandas: 2.2.1
6. Numpy: 1.26.4
7. Sklearn: 1.4.1.post1
8. Scipy: 1.15.2
9. Matplotlib: 3.10.1
10. Seaborn: 0.12.2
11. Sentencepiece: 0.1.99
12. Torch: 2.8.0+cu128

**Table 3.** Effect of *SCL2205*$_s$ and *DEEP-TV*$_s$ versus independent test sets processing, before comparing the performance of the datasets. **Original**: Full independent test set size. **No overlap**: Samples remaining after overlap reduction with the training datasets. **Stats valid**: Samples remaining after ensuring validity for McNemar's and bootstrapping CI paired statistical comparison.

| | ***SCL2205***$_s$ | | | | | |
|---|---|---|---|---|---|---|
| **Subcellular** | **DEEP-SS** | | | **DEEP-HPA** | | |
| **location** | **Original** | **No overlap** | **Stats valid** | **Original** | **No overlap** | **Stats valid** |
| Secreted‡ | 522 | 184 | 158 | NA | NA | NA |
| Membrane | 170 | 108 | 90 | 161 | 149 | 145 |
| Mitochondrion | 111 | 73 | 64 | 175 | 153 | 149 |
| Peroxisome | 77 | 30 | 24 | 7 | 7 | 7 |
| Nucleus | 63 | 43 | 39 | 664 | 622 | 602 |
| Cytoplasm | 10 | 6 | 6 | 263 | 248 | 238 |
| ER | 5 | 4 | 4 | 31 | 29 | 27 |
| Plastid‡ | 1 | 0 | 0 | NA | NA | NA |
| **Totals** | 958 | 448 | 385 | 1301 | 1208 | 1168 |
| **SwissProt train-validation (*DEEP-TV*)**$_s$ | | | | | | |
| Secreted‡ | 522 | 218 | 158 | NA | NA | NA |
| Membrane | 170 | 114 | 90 | 161 | 149 | 145 |
| Mitochondrion | 111 | 77 | 64 | 175 | 163 | 149 |
| Peroxisome | 77 | 37 | 24 | 7 | 7 | 7 |
| Nucleus | 63 | 44 | 39 | 664 | 627 | 602 |
| Cytoplasm | 10 | 7 | 6 | 263 | 244 | 238 |
| ER | 5 | 4 | 4 | 31 | 27 | 27 |
| Plastid‡ | 1 | 0 | 0 | NA | NA | NA |
| **Totals** | 958 | 501 | 385 | 1301 | 1217 | 1168 |

‡ Secreted and Plastid classes absent in **DEEP-HPA**.

# Supplementary Experiments

## Model architectures

**Tokeniser training: SentencePiece**

```
sentencepiece.set_random_generator_seed(2023)
sentencepiece.SentencePieceTrainer.train(input=<input_corpus>,
model_prefix=<output_name>,
vocab_size=15000,
normalization_rule_name='nfkc',
max_sentence_length=4924,
max_sentencepiece_length=9,
bos_id=-1,
eos_id=-1,
character_coverage=1.0,
add_dummy_prefix=False,
num_sub_iterations=3)
```

Embedder network: CNN architecture

```
class CNN(nn.Module):
    def __init__(self, pool_window: int, pool_stride: int, pooled_dim: int, num_targets: int, seq_len: int):
        super().__init__()
        self.pool_window = pool_window
        self.pool_stride = pool_stride
        self.pooled_dim = pooled_dim
        self.num_targets = num_targets
        self.seq_len = seq_len

        self.cnn_l1 = nn.Sequential(
            nn.Conv1d(in_channels=1, out_channels=20, kernel_size=21,
                      padding='same', device=device),
            nn.ReLU())
        self.cnn_l2 = nn.Sequential(
            nn.Conv1d(in_channels=1, out_channels=20, kernel_size=9,
                      padding='same', device=device),
            nn.ReLU())
        self.cnn_l3 = nn.Sequential(
            nn.Conv1d(in_channels=1, out_channels=20, kernel_size=3,
                      padding='same', device=device),
            nn.ReLU())

        self.cnn_l4 = nn.Sequential(
            nn.Conv1d(in_channels=60, out_channels=30, kernel_size=3,
                      padding='same', device=device),
            nn.ReLU(),
            nn.Dropout())

        self.max_pool = nn.AvgPool1d(kernel_size=pool_window, stride=pool_stride)
        self.layer_flattener = nn.Flatten()

        self.fc = nn.Sequential(
            nn.Linear(in_features=30 * pooled_dim, out_features=256, device=device),
            nn.ReLU(),
            nn.Linear(256, 128, device=device),
            nn.ReLU(),
            nn.Linear(128, 64, device=device),
            nn.ReLU(),
            nn.Linear(64, num_targets, device=device),
        )

    def forward(self, x):
        learned_motifs = list(map(lambda y:y(x), [self.cnn_l1, self.cnn_l2, self.cnn_l3]))
        concat_motifs = torch.concat(learned_motifs, dim=1)
        x = self.cnn_l4(concat_motifs)
        x = self.max_pool(x)
        x = self.layer_flattener(x)
        logits = self.fc(x)
        return logits
```

PLM Embedding function: ProtT5

```
def chunk_embed_batch(tokeniser, model, seqs, max_len=512, overlap=64, device=None):
    '''
    Embed a batch of protein sequences using ProtT5 with chunking.
    Each sequence may be longer than max_len, so we split it into overlapping chunks.

    Args:
        tokeniser: ProtT5 tokeniser
        model: ProtT5 encoder model
        seqs (list[str]): List of sequences in this batch
        max_len (int): Max token length per chunk (ProtT5 default = 512)
        overlap (int): Overlap between consecutive chunks
        device (str): 'cuda' or 'cpu'

    Returns:
        np.ndarray: (len(seqs), hidden_size) mean-pooled embedding per sequence
    '''
    step = max_len - overlap

    # record chunk to sequence mapping
    chunk_texts, chunk_to_seq = [], []
    for seq_idx, seq in enumerate(seqs):
        chunks = [seq[i:i+max_len] for i in range(0, len(seq), step)]
        chunk_texts.extend(chunks)
        chunk_to_seq.extend([seq_idx] * len(chunks)) #expand index

    model_device = next(model.parameters()).device # model device

    # tokenise all chunks in this batch
    inputs = tokenizer(chunk_texts, return_tensors='pt', padding='longest',)

    inputs = {k: v.to(device) for k, v in inputs.items()}
    input_ids = inputs['input_ids'].to(model_device if not device else device)
    attention_mask = inputs['attention_mask'].to(model_device if not device else device)

    # optionally trim to MAX_SEQ_LEN where it is less than the model's (512)
    if input_ids.size(1) > max_len:
        input_ids = input_ids[:, :max_len]
        attention_mask = attention_mask[:, :max_len]

    # check device locations
    if model_device != input_ids.device:
        raise RuntimeError(f'Model is on {model_device}, but inputs are on {input_ids.device}')

    with torch.no_grad():
        outputs = model(input_ids=input_ids, attention_mask=attention_mask)
        last_hidden = outputs.last_hidden_state  # (batch_chunks, seq_len, hidden_size)

        # mean-pool per chunk
        emb = (last_hidden * attention_mask.unsqueeze(-1)).sum(1) / attention_mask.sum(1).unsqueeze(-1)
        emb = emb.cpu().numpy()

    # aggregate chunks to per-sequence
    hidden_size = emb.shape[1]
    seq_embeddings = np.zeros((len(seqs), hidden_size), dtype=np.float32)

    for seq_idx in range(len(seqs)):
        mask = np.array(chunk_to_seq) == seq_idx
        seq_embeddings[seq_idx] = emb[mask].mean(axis=0)

    return seq_embeddings # (N, hidden_size)
```

PLM Embedding function call

```
# config
MODEL_NAME = 'Rostlab/prot_t5_xl_half_uniref50-enc' if device == 'cpu' else 'Rostlab/prot_t5_xl_uniref50'

# load tokeniser & model
tokeniser = T5Tokenizer.from_pretrained(MODEL_NAME, do_lower_case=False, legacy=False)
model_prot_t5 = T5EncoderModel.from_pretrained(MODEL_NAME)
model_prot_t5 = model_prot_t5.to(device)

# if using half precision and GPU is available, cast to fp16 for memory speed-ups
use_fp16 = (device.startswith('cuda'))
if use_fp16:
    model_prot_t5 = model_prot_t5.half()

model_prot_t5.eval()

# generate embeddings
emb = chunk_embed_batch(tk_model=tokeniser, emb_model=model_prot_t5, batch=64, max_len=512, overlap=32, device=device)
```

Classification network: MLP architecture

```
class FFN(nn.Module):
    def __init__(self, emb_size: int, num_targets: int, device):
        super().__init__()
        self.emb_size = emb_size
        self.num_targets = num_targets
        self.device = device

        self.layer_flattener = nn.Flatten()

        self.fc = nn.Sequential(
            nn.Linear(in_features=self.emb_size, out_features=256, device=self.device),
            nn.ReLU(),
            nn.Linear(256, 128, device=self.device),
            nn.ReLU(),
            nn.Linear(128, 64, device=self.device),
            nn.ReLU(),
            nn.Linear(64, self.num_targets, device=self.device),
            )

    def forward(self, x):
        x = self.layer_flattener(x)
        logits = self.fc(x)
        return logits
```

# Supplementary Results

## Mapping ablation performance

**Table 4.** Mapping performance by subcellular location for DEEP-SS and DEEP-HPA. While we observed a remarkable improvement in label mapping for SwissProt sorting signal (*DEEP-SS*) (9%), the improvement for human protein atlas (*DEEP-HPA*) was marginal.

| **Subcellular location** | **Baseline Prevalence** | **Label Mapping** | **No Label Mapping** |
|---|---|---|---|
| | **DEEP-SS** | | |
| Macro PR-AUC* | 0.143 | 0.296 | 0.206 |
| Secreted | 0.410 | 0.777 | 0.788 |
| Nucleus | 0.096 | 0.555 | 0.242 |
| Membrane | 0.241 | 0.391 | 0.170 |
| Mitochondrion | 0.165 | 0.243 | 0.161 |
| Peroxisome | 0.067 | 0.070 | 0.053 |
| ER | 0.009 | 0.021 | 0.010 |
| Cytoplasm | 0.013 | 0.015 | 0.016 |
| | **DEEP-HPA** | | |
| Macro PR-AUC* | 0.167 | 0.205 | 0.190 |
| Secreted | NA | NA | NA |
| Nucleus | 0.514 | 0.584 | 0.568 |
| Membrane | 0.123 | 0.134 | 0.134 |
| Mitochondrion | 0.127 | 0.206 | 0.160 |
| Peroxisome | 0.006 | 0.011 | 0.010 |
| ER | 0.024 | 0.036 | 0.033 |
| Cytoplasm | 0.206 | 0.260 | 0.233 |

* Overall metric area under the precision–recall curve (PR-AUC). We computed the average prevalence across classes.

## Convolutional neural network (CNN) model performance

**Table 5.** *SCL2205$_s$* performance benchmarking against *DEEP-TV$_s$* using two external datasets (*DEEP-SS* and *DEEP-HPA*) based on CNN models. Although *DEEP-TV$_s$* exhibits better discriminative ability (PR-AUC) than *SCL2205* – by 7.7% points on *DEEP-SS* – the difference is marginal for *DEEP-HPA*.

| Subcellular location | Baseline Prevalence | *SCL2205$_s$* Eight-class | *DEEP-TV$_s$* Eight-class |
|---|---|---|---|
| **DEEP-SS** | | | |
| **Macro PR-AUC*** | 0.143 | 0.257 | 0.334 |
| Secreted | 0.410 | 0.657 | 0.723 |
| Membrane | 0.234 | 0.384 | 0.353 |
| Mitochondrion | 0.166 | 0.356 | 0.315 |
| Nucleus | 0.101 | 0.291 | 0.737 |
| Peroxisome | 0.062 | 0.074 | 0.096 |
| ER | 0.010 | 0.023 | 0.098 |
| Cytoplasm | 0.016 | 0.014 | 0.014 |
| Plastid | NA | NA | NA |
| **DEEP-HPA** | | | |
| **Macro PR-AUC*** | 0.167 | 0.194 | 0.184 |
| Secreted† | NA | NA | NA |
| Membrane | 0.124 | 0.135 | 0.127 |
| Mitochondrion | 0.128 | 0.147 | 0.147 |
| Nucleus | 0.515 | 0.576 | 0.603 |
| Peroxisome | 0.006 | 0.045 | 0.006 |
| ER | 0.023 | 0.029 | 0.025 |
| Cytoplasm | 0.204 | 0.231 | 0.198 |
| Plastid† | NA | NA | NA |

* Overall metric PR-AUC. We computed the average prevalence across classes.

† Category missing in dataset.

## Protein language model (PLM) model performance

**Table 6.** *SCL2205*$_s$ performance benchmarking against *DEEP-TV*$_s$ using two external datasets (*DEEP-SS* and *DEEP-HPA*) based on PLMs. Although *SCL2205* exhibits better discriminative ability (PR-AUC) than *DEEP-TV*$_s$ – by 10.8% points on *DEEP-SS* – the difference is marginal for *DEEP-HPA*.

| **Subcellular location** | **Baseline Prevalence** | ***SCL2205*$_s$ Eight-class** | ***DEEP-TV*$_s$ Eight-class** |
|---|---|---|---|
| **DEEP-SS** | | | |
| **Macro PR-AUC*** | 0.143 | 0.561 | 0.453 |
| Secreted | 0.410 | 0.895 | 0.798 |
| Membrane | 0.234 | 0.876 | 0.751 |
| Mitochondrion | 0.166 | 0.953 | 0.975 |
| Nucleus | 0.101 | 0.959 | 0.557 |
| Peroxisome | 0.062 | 0.197 | 0.060 |
| ER | 0.010 | 0.020 | 0.012 |
| Cytoplasm | 0.016 | 0.023 | 0.021 |
| Plastid† | NA | NA | NA |
| **DEEP-HPA** | | | |
| **Macro PR-AUC*** | 0.167 | 0.345 | 0.359 |
| Secreted† | NA | NA | NA |
| Membrane | 0.124 | 0.430 | 0.489 |
| Mitochondrion | 0.128 | 0.594 | 0.643 |
| Nucleus | 0.515 | 0.818 | 0.761 |
| Peroxisome | 0.006 | 0.012 | 0.030 |
| ER | 0.023 | 0.037 | 0.037 |
| Cytoplasm | 0.204 | 0.180 | 0.193 |
| Plastid† | NA | NA | NA |

* Overall metric PR-AUC. We computed the average prevalence across classes for each test set.
† Category missing in dataset.

## **SCL2205** final composition summaries

### *Training-validation-testing stream composition*

**Table 7.** Subcellular location counts across dataset splits.

| Subcellular location | Original | Training | Validation | Testing* |
|---|---|---|---|---|
| Membrane | 4718 | 3837 | 277 | 604 |
| Nucleus | 4179 | 3202 | 329 | 648 |
| Secreted | 2635 | 2245 | 131 | 259 |
| Cytoplasm | 2465 | 1922 | 166 | 377 |
| Cytoplasm;Nucleus | 2282 | 1796 | 160 | 326 |
| Mitochondrion | 946 | 740 | 67 | 139 |
| Plastid | 587 | 467 | 37 | 83 |
| Centrosome;Cytoplasm;Cytoskeleton;MTOC** | 308 | 228 | 27 | 53 |
| Cytoplasm;Membrane | 242 | 190 | 17 | 35 |
| Cytoplasm;Cytoskeleton | 215 | 162 | 15 | 38 |
| ER | 185 | 146 | 14 | 25 |
| Cell projection | 176 | 128 | 16 | 32 |
| Peroxisome | 136 | 120 | 4 | 12 |
| **Totals** | **19074** | **15183** | **1260** | **2631** |

* The *Testing* split is common across *cross-validation-testing* (CVT) and *training-validation-testing* (TVT) streams.
** For brevity, Microtubule organizing center (MTOC).

### *Cross-validation-testing stream composition*

Table 8: Subcellular location counts for Train, Validation, and Test splits across five cross-validation folds.

| Subcellular location | Training | Validation | Testing* |
|---|---|---|---|
| **Fold 0** | | | |
| Membrane | 3804 | 310 | 604 |
| Nucleus | 3214 | 317 | 648 |
| Secreted | 2257 | 119 | 259 |
| Cytoplasm | 1916 | 172 | 377 |
| Cytoplasm;Nucleus | 1806 | 150 | 326 |
| Mitochondrion | 744 | 63 | 139 |
| Plastid | 475 | 29 | 83 |
| Centrosome;Cytoplasm;Cytoskeleton;MTOC** | 228 | 27 | 53 |
| Cytoplasm;Membrane | 194 | 13 | 35 |
| Cytoplasm;Cytoskeleton | 161 | 16 | 38 |
| ER | 144 | 16 | 25 |
| Cell projection | 127 | 17 | 32 |
| Peroxisome | 117 | 7 | 12 |
| **Totals** | **15187** | **1256** | **2631** |
| **Fold 1** | | | |
| Membrane | 3836 | 278 | 604 |
| Nucleus | 3217 | 314 | 648 |
| Secreted | 2241 | 135 | 259 |
| Cytoplasm | 1927 | 161 | 377 |
| Cytoplasm;Nucleus | 1784 | 172 | 326 |
| Mitochondrion | 751 | 56 | 139 |
| Plastid | 474 | 30 | 83 |
| Centrosome;Cytoplasm;Cytoskeleton;MTOC** | 223 | 32 | 53 |
| Cytoplasm;Membrane | 191 | 16 | 35 |
| Cytoplasm;Cytoskeleton | 161 | 16 | 38 |
| ER | 153 | 7 | 25 |
| Cell projection | 128 | 16 | 32 |
| Peroxisome | 117 | 7 | 12 |

*Continued on next page*

| Subcellular location | Train | Validation | Test |
|---|---|---|---|
| **Totals** | **15203** | **1240** | **2631** |
| | **Fold 2** | | |
| Membrane | 3819 | 295 | 604 |
| Nucleus | 3209 | 322 | 648 |
| Secreted | 2257 | 119 | 259 |
| Cytoplasm | 1918 | 170 | 377 |
| Cytoplasm;Nucleus | 1790 | 166 | 326 |
| Mitochondrion | 740 | 67 | 139 |
| Plastid | 467 | 37 | 83 |
| Centrosome;Cytoplasm;Cytoskeleton;MTOC** | 231 | 24 | 53 |
| Cytoplasm;Membrane | 190 | 17 | 35 |
| Cytoplasm;Cytoskeleton | 165 | 12 | 38 |
| ER | 148 | 12 | 25 |
| Cell projection | 131 | 13 | 32 |
| Peroxisome | 120 | 4 | 12 |
| **Totals** | **15185** | **1258** | **2631** |
| | **Fold 3** | | |
| Membrane | 3817 | 297 | 604 |
| Nucleus | 3227 | 304 | 648 |
| Secreted | 2256 | 120 | 259 |
| Cytoplasm | 1926 | 162 | 377 |
| Cytoplasm;Nucleus | 1796 | 160 | 326 |
| Mitochondrion | 741 | 66 | 139 |
| Plastid | 469 | 35 | 83 |
| Centrosome;Cytoplasm;Cytoskeleton;MTOC** | 228 | 27 | 53 |
| Cytoplasm;Membrane | 190 | 17 | 35 |
| Cytoplasm;Cytoskeleton | 161 | 16 | 38 |
| ER | 149 | 11 | 25 |
| Cell projection | 131 | 13 | 32 |
| Peroxisome | 119 | 5 | 12 |
| **Totals** | **15210** | **1233** | **2631** |
| | **Fold 4** | | |
| Membrane | 3861 | 253 | 604 |
| Nucleus | 3221 | 310 | 648 |
| Secreted | 2280 | 96 | 259 |
| Cytoplasm | 1922 | 166 | 377 |
| Cytoplasm;Nucleus | 1814 | 142 | 326 |
| Mitochondrion | 739 | 68 | 139 |
| Plastid | 461 | 43 | 83 |
| Centrosome;Cytoplasm;Cytoskeleton;MTOC** | 227 | 28 | 53 |
| Cytoplasm;Membrane | 193 | 14 | 35 |
| Cytoplasm;Cytoskeleton | 161 | 16 | 38 |
| ER | 143 | 17 | 25 |
| Cell projection | 127 | 17 | 32 |
| Peroxisome | 116 | 8 | 12 |
| **Totals** | **15265** | **1178** | **2631** |

* The *Testing* split is common across CVT and TVT streams.
** For brevity, Microtubule organizing center (MTOC).

## Cochran‘s Q test

Cochran's Q test was used to assess the inductive biases between the two model architectures used to evaluate *SCL2205* and *DEEP-TV*. Based on the stratified bootstrap deltas of the PR-AUC between the models, the following were the results. For the *DEEP-SS*: $Q = 53.06$, $p_{het} = 3.24 \times 10^{-13}$; *DEEP-HPA*: $Q = 268.5$, $p_{het} < 0.001$. The observation supports that models A and B retrieved heterogeneous signals from the two datasets.

## References

Fu L et al. Sequence analysis cd-hit: accelerated for clustering the next-generation sequencing data. *Bioinformatics*, 28:3150–3152, 12 2012. doi: 10.1093/bioinformatics/bts565.
