# Supplementary material for: Developing SCL2205: a protein sequence-based spatial modelling dataset for the protein language model frontier
Source: Bioinformatics. 2026 Sep 18;42(9):btag666. doi: 10.1093/bioinformatics/btag666 (PMC13616090; doi:10.1093/bioinformatics/btag666)
Supplement: Supplementary file 1 [file btag666_supplementary_data.zip › supplementary-file-s2.pdf]

Manual Subcellular Location Mapping and Harmonisation

Table 1: **Manual subcellular location mapping and harmonisation**. The columns comprise the mined original subcellular locations, their corresponding counts, and the mapped/harmonised annotations, respectively. The exercise was guided by the universal protein knowledgebase (UniProtKB)'s subcellular localisation (SCL) ontology information (UniProtConsortium et al. 2023) and the interactive cell from *SwissBioPics* library (Mercier et al. 2022).

Location	Counts	Mapped
Secreted	4877	NA
Nucleus	3695	NA
Cytoplasm	2876	NA
Cytoplasm;Nucleus	2350	NA
Mitochondrion	955	NA
Membrane	324	NA
Cytoplasm;Cytoskeleton	253	NA
Endoplasmic reticulum	150	NA
Peroxisome	145	NA
Centrosome;Cytoplasm;Cytoskeleton;Microtubule organizing center	96	NA
Cytoplasm;Membrane	92	NA
Cell projection	6	NA
Plastid	6	NA
Cell membrane	2310	Membrane
Endoplasmic reticulum membrane	888	Membrane
Cell inner membrane	602	Membrane
Nucleolus;Nucleus	486	Nucleus
Mitochondrion inner membrane	463	Membrane
Chloroplast;Plastid	395	Plastid
Cytoplasm;Cytosol	357	Cytoplasm
Golgi apparatus membrane	314	Membrane
Chromosome;Nucleus	313	Nucleus
Cell wall;Secreted	301	Secreted
Mitochondrion matrix	216	Mitochondrion
Cell membrane;Cytoplasm	200	Cytoplasm;Membrane
Vacuole membrane	193	Membrane
Chloroplast thylakoid membrane;Plastid	169	Membrane
Cytoplasm;Cytosol;Nucleus	158	Cytoplasm;Nucleus
Chloroplast stroma;Plastid	140	Plastid
Cellular thylakoid membrane	131	Membrane
Mitochondrion outer membrane	124	Membrane
Cell outer membrane	124	Membrane
Extracellular matrix;Extracellular space;Secreted	113	Secreted
Cytoplasm;Nucleolus;Nucleus	112	Cytoplasm;Nucleus
Apical cell membrane	112	Membrane
Host nucleus	109	Nucleus
Mitochondrion membrane	93	Membrane
Extracellular space;Secreted	87	Secreted
Cytoplasm;Perinuclear region	82	Cytoplasm
Host cytoplasm	81	Cytoplasm
Nucleoplasm;Nucleus	80	Nucleus

Location	Counts	Mapped
Endoplasmic reticulum membrane;Golgi apparatus membrane	78	Membrane
Host cytoplasm;Host nucleus	76	Cytoplasm;Nucleus
Peroxisome membrane	76	Membrane
Nucleus speckle	75	Nucleus
Endoplasmic reticulum lumen	72	ER
Cell membrane;Endoplasmic reticulum membrane	72	Membrane
Cell projection;Cilium	71	Cell projection
Cell projection;Cilium;Flagellum	68	Cell projection
Lysosome membrane	66	Membrane
Centriole;Centrosome;Cytoplasm;Cytoskeleton;Microtubule organizing center	64	Centrosome;Cytoplasm;Cytoskeleton;Microtubule organizing center
Basolateral cell membrane	56	Membrane
Cell membrane;Membrane raft	55	Membrane
Nucleus;Nucleus speckle	51	Nucleus
Endosome membrane	49	Membrane
Cell cortex;Cytoplasm	46	Cytoplasm
Apical cell membrane;Cell membrane	46	Membrane
Nucleus membrane	44	Membrane
Microsome membrane	43	Membrane
Cytoplasm;Nucleus;Perinuclear region	42	Cytoplasm;Nucleus
Cytoplasm;Nucleoplasm;Nucleus	41	Cytoplasm;Nucleus
Chloroplast thylakoid lumen;Plastid	40	Plastid
Nucleolus;Nucleoplasm;Nucleus	37	Nucleus
Nucleus envelope	36	Membrane
Cytoplasm;Cytoskeleton;Microtubule organizing center;Spindle pole body	34	Centrosome;Cytoplasm;Cytoskeleton;Microtubule organizing center
Basolateral cell membrane;Cell membrane	34	Membrane
Nucleus inner membrane	32	Membrane
Endoplasmic reticulum membrane;Microsome membrane	32	Membrane
Endoplasmic reticulum membrane;Nucleus membrane	29	Membrane
Chloroplast;Plastid;Plastoglobule	29	Plastid
Nucleoplasm;Nucleus;Nucleus speckle	28	Nucleus
Cell membrane;Golgi apparatus membrane	26	Membrane
Cytoplasmic vesicle membrane	26	Membrane
Cell projection;Dendrite	25	Cell projection
Centrosome;Cytoplasm;Cytoskeleton;Microtubule organizing center;Nucleus	25	Centrosome;Cytoplasm;Cytoskeleton;Microtubule organizing center
Cell membrane;Postsynaptic cell membrane	25	Membrane
Cell membrane;Vacuole membrane	25	Membrane
Host cell membrane	25	Membrane
Centrosome;Cytoplasm;Cytoskeleton;Microtubule organizing center;Spindle	24	Centrosome;Cytoplasm;Cytoskeleton;Microtubule organizing center
Early endosome membrane	24	Membrane
Cell membrane;Cytoplasmic vesicle membrane	24	Membrane
Centriole;Centrosome;Cilium basal body; Cytoplasm;Cytoskeleton;Microtubule organizing center	23	Centrosome;Cytoplasm;Cytoskeleton;Microtubule organizing center
Chloroplast outer membrane;Plastid	23	Membrane
Cell membrane;Endosome membrane	23	Membrane
Preautophagosomal structure membrane	23	Membrane

Location	Counts	Mapped
Chloroplast thylakoid;Plastid	23	Plastid
Axon;Cell projection	22	Cell projection
Cytoplasm;Nucleolus;Nucleoplasm;Nucleus	22	Cytoplasm;Nucleus
Late endosome membrane;Lysosome membrane	22	Membrane
Endomembrane system	22	Membrane
Nucleus;Pml body	22	Nucleus
Cytoplasm;Nucleus;P-body	20	Cytoplasm;Nucleus
Cytoplasm;Cytosol;Membrane	19	Cytoplasm;Membrane
Late endosome membrane	19	Membrane
Host endoplasmic reticulum membrane	16	Membrane
Cellular chromatophore membrane	16	Membrane
Nucleus matrix	16	Nucleus
Peroxisome matrix	16	Peroxisome
Sarcoplasmic reticulum membrane	15	Membrane
Cell outer membrane;Cell surface	15	Membrane
Cell projection;Stereocilium	14	Cell projection
Cytoplasm;Cytoskeleton;Microtubule organizing center;Nucleus; Spindle pole body	14	Centrosome;Cytoplasm;Cytoskeleton;Microtubule organizing center
Centriolar satellite;Centrosome;Cytoplasm;Cytoskeleton; Microtubule organizing center	13	Centrosome;Cytoplasm;Cytoskeleton;Microtubule organizing center
Postsynaptic cell membrane	13	Membrane
Chloroplast nucleoid;Chloroplast stroma;Plastid	13	Plastid
Cell cortex;Cytoplasm;Nucleus	12	Cytoplasm;Nucleus
Apical cell membrane;Basolateral cell membrane	12	Membrane
Endoplasmic reticulum membrane;Vacuole membrane	12	Membrane
Centrosome;Cytoplasm;Cytoskeleton;Microtubule organizing center;Spindle pole	11	Centrosome;Cytoplasm;Cytoskeleton;Microtubule organizing center
Cytoplasm;Cytoplasmic granule	11	Cytoplasm
Host cytoplasm;Host perinuclear region	11	Cytoplasm
Cytoplasm;Cytoplasmic granule;Perinuclear region	11	Cytoplasm
Cell cortex;Cytoplasm;Cytoskeleton	11	Cytoplasm;Cytoskeleton
Cytoplasm;Cytoskeleton;Spindle;Spindle pole	11	Cytoplasm;Cytoskeleton
Cell membrane;Membrane	11	Membrane
Recycling endosome membrane	11	Membrane
Cajal body;Nucleus	11	Nucleus
Endoplasmic reticulum membrane;Mitochondrion membrane	10	Membrane
Membrane raft	10	Membrane
Cell membrane;Lysosome membrane	10	Membrane
Endosome membrane;Lysosome membrane	10	Membrane
Mitochondrion matrix;Mitochondrion nucleoid	10	Mitochondrion
Host nucleolus;Host nucleus	10	Nucleus
Cytoplasm;Nucleus;Pml body	9	Cytoplasm;Nucleus
Endosome membrane;Golgi apparatus membrane	9	Membrane
Centrosome;Cytoplasm;Cytoskeleton;Microtubule organizing center;Spindle;Spindle pole	7	Centrosome;Cytoplasm;Cytoskeleton;Microtubule organizing center
Cytoplasm;Nucleus;P-body;Stress granule	7	Cytoplasm;Nucleus
Cell membrane;Endosome membrane;Lysosome membrane	7	Membrane
Cytoplasm;Nucleoplasm;Nucleus;Nucleus speckle	6	Cytoplasm;Nucleus
Endoplasmic reticulum membrane;Membrane	6	Membrane

Location	Counts	Mapped
Cajal body;Nucleus;Nucleus speckle	6	Nucleus
Chloroplast stroma;Chloroplast thylakoid membrane;Plastid	6	Plastid
Centrosome;Cytoplasm;Cytoskeleton;Cytosol;Microtubule organizing center	5	Centrosome;Cytoplasm;Cytoskeleton;Microtubule organizing center
Centriolar satellite;Centriole;Centrosome;Cytoplasm; Cytoskeleton;Microtubule organizing center	5	Centrosome;Cytoplasm;Cytoskeleton;Microtubule organizing center
Centrosome;Cytoplasm;Cytoskeleton;Microtubule organizing center;Nucleus;Spindle	5	Centrosome;Cytoplasm;Cytoskeleton;Microtubule organizing center
Centrosome;Cilium basal body;Cytoplasm;Cytoskeleton; Microtubule organizing center	5	Centrosome;Cytoplasm;Cytoskeleton;Microtubule organizing center
Centromere;Centrosome;Chromosome;Cytoplasm;Cytoskeleton; Kinetochore;Microtubule organizing center;Spindle	5	Centrosome;Cytoplasm;Cytoskeleton;Microtubule organizing center
Cytoplasm;Cytosol;Nucleolus;Nucleus	5	Cytoplasm;Nucleus
Host endoplasmic reticulum	5	ER
Early endosome membrane;Recycling endosome membrane	5	Membrane
Golgi apparatus membrane;Vacuole membrane	5	Membrane
Endosome membrane;Vacuole membrane	5	Membrane
Amyloplast;Plastid	5	Plastid
Centrosome;Chromosome;Cytoplasm;Cytoskeleton;Microtubule organizing center;Nucleus	4	Centrosome;Cytoplasm;Cytoskeleton;Microtubule organizing center
Centriole;Centrosome;Cytoplasm;Cytoskeleton;Microtubule organizing center;Nucleus	4	Centrosome;Cytoplasm;Cytoskeleton;Microtubule organizing center
Cytoplasm;Cytoskeleton;Cytosol	4	Cytoplasm;Cytoskeleton
Nucleolus;Nucleoplasm;Nucleus;Nucleus speckle	4	Nucleus
Cytoplasm;Cytosol;Nucleoplasm;Nucleus	3	Cytoplasm;Nucleus
Cytoplasm;Nucleoplasm;Nucleus;Perinuclear region	3	Cytoplasm;Nucleus
Cell membrane;Multi-pass membrane protein	3	Membrane
Early endosome membrane;Golgi apparatus membrane	3	Membrane
Cell membrane;Recycling endosome membrane	3	Membrane
Cajal body;Nucleolus;Nucleus	3	Nucleus
Cytoplasmic vesicle;Extracellular space;Secreted;Secretory vesicle	3	Secreted
Centrosome;Cytoplasm;Cytoskeleton;Microtubule organizing center;Nucleolus;Nucleoplasm;Nucleus	2	Centrosome;Cytoplasm;Cytoskeleton;Microtubule organizing center
Host cytoplasm;Host cytosol	2	Cytoplasm
Cytoplasm;Nucleoplasm;Nucleus;Nucleus matrix	2	Cytoplasm;Nucleus
Cajal body;Cytoplasm;Nucleus	2	Cytoplasm;Nucleus
Cajal body;Cytoplasm;Nucleoplasm;Nucleus	2	Cytoplasm;Nucleus
Cajal body;Cytoplasm;Nucleus;Nucleus speckle	2	Cytoplasm;Nucleus
Cytoplasm;Nucleus;Nucleus speckle;P-body;Pml body	2	Cytoplasm;Nucleus
Cytoplasm;Nucleolus;Nucleoplasm;Nucleus;Nucleus speckle	2	Cytoplasm;Nucleus
Cytoplasm;Nucleoplasm;Nucleus;Pml body	2	Cytoplasm;Nucleus
Cytoplasm;Nucleus;Perikaryon	2	Cytoplasm;Nucleus
Sarcoplasmic reticulum	2	ER
Cell membrane;Endomembrane system;Endosome membrane	2	Membrane
Glyoxysome membrane;Peroxisome membrane	2	Membrane
Cell membrane;Prospore membrane	2	Membrane
Apical cell membrane;Membrane	2	Membrane
Cell inner membrane;Cell outer membrane	2	Membrane
Nucleus envelope;Nucleus membrane	2	Membrane

Location	Counts	Mapped
Nucleoplasm;Nucleus;Pml body	2	Nucleus
Chromoplast;Plastid	2	Plastid
Chromosome;Cytoplasm;Nucleus;Pml body	1	Cytoplasm;Nucleus
Cytoplasm;Cytoplasmic vesicle;Nucleus;Pml body	1	Cytoplasm;Nucleus

References

Mercier P. L et al. Swissbiopics—an interactive library of cell images for the visualization of subcellular location data. *Database: The Journal of Biological Databases and Curation*, 2022:1–5, 2022. ISSN 17580463. doi: 10.1093/DATABASE/BAAC026.

UniProtConsortium et al. Uniprot: the universal protein knowledgebase in 2023. *Nucleic Acids Research*, 51:D523–D531, 1 2023. ISSN 0305-1048. doi: 10.1093/NAR/GKAC1052.
